# Phase III trial of sodium dichloroacetate for pyruvate dehydrogenase complex deficiency in children

**DOI:** 10.1172/jci.insight.200149

**Published:** 2026-09-22

**Authors:** Peter W. Stacpoole, Jose E. Abdenur, Jirair K. Bedoyan, Lorenzo Botto, Gregory M. Enns, Marni J. Falk, Rebecca Ganetzky, Cheryl Garganta, Kevin Glinton, Andrea Gropman, Sharon Hamm, Eugenia Henry, Nicola Longo, Richard Neiberger, Russell P. Saneto, Fernando Scaglia, Sub H. Subramony, Jerry Vockley, Richard E. Wagner

**Affiliations:** 1 Department of Medicine and Department of Biochemistry and Molecular Biology, University of Florida College of Medicine, Gainesville, Florida, USA.; 2 Children’s Hospital of Orange County, Orange, California, USA.; 3 Department of Pediatrics, University of Pittsburgh School of Medicine, and UPMC Children’s Hospital of Pittsburgh, Pittsburgh, Pennsylvania, USA.; 4 Department of Pediatrics, University of Utah School of Medicine, Salt Lake City, Utah, USA.; 5 Department of Pediatrics, Stanford University School of Medicine, Stanford, California, USA.; 6 Mitochondrial Medicine Frontier Program, Children’s Hospital of Philadelphia, Philadelphia, Pennsylvania, USA.; 7 Division of Human Genetics, Department of Pediatrics, and University of Pennsylvania Perelman School of Medicine, Philadelphia, Pennsylvania, USA.; 8 Department of Pediatrics, University of Florida College of Medicine, Gainesville, Florida, USA.; 9 Department of Molecular and Human Genetics, Baylor College of Medicine and Texas Children’s Hospital, Houston, Texas, USA.; 10 Department of Genomic and Translational Neuroscience, St. Jude Children’s Research Hospital, Memphis, Tennessee, USA.; 11 Saol Therapeutics, Roswell, Georgia, USA.; 12 Firma Clinical Research, Elk Grove Village, Illinois, USA.; 13 Division of Clinical Genetics/Human Genetics, UCLA, Los Angeles, California, USA.; 14 Norcliffe Foundation Center for Integrative Brain Research, Seattle Children’s Hospital, Seattle, Washington, USA.; 15 Division of Pediatric Neurology, Department of Neurology, University of Washington, Seattle, Washington, USA.; 16 Joint BCM-CUHK Center of Medical Genetics, Prince of Wales Hospital, ShaTin, Hong Kong.; 17 Baylor Genetics, Houston, Texas, USA.; 18 Department of Neurology, University of Florida College of Medicine, Gainesville, Florida, USA.; 19 Medosome Biotec, LLC, Alachua, Florida, USA.

**Keywords:** Clinical Research, Metabolism, Clinical trials, Drug therapy, Mitochondria

## Abstract

**BACKGROUND:**

Dichloroacetate (DCA) is an orally administered structural analog of pyruvate, an endogenous pyruvate dehydrogenase kinase inhibitor.

**METHODS:**

We conducted a phase III multicenter trial in 34 children with pyruvate dehydrogenase complex deficiency (PDCD). Participants were randomly allocated to 4 months of treatment with DCA or a placebo, followed by a 1-month washout period and crossover to the alternate arm, and could continue into an open-label extension period. DCA dosing was predetermined by pharmacogenomic analysis of *GSTZ1*, which modulates DCA metabolism. The primary endpoint was the observer-reported outcomes motor domain (ObsRO_motor_) score. Additional assessments evaluated motor function, plasma lactate levels, and survival.

**RESULTS:**

Chronic DCA was well tolerated and safe. The primary endpoint, ObsRO_motor_, was not statistically significantly different between the treatment and placebo groups (*P* = 0.512). However, longer-term treatment, including the open-label extension, showed a statistically significant treatment effect (*P* = 0.002), especially in participants with higher baseline motor impairment (ObsRO_motor_ ≥ 8; *P* = 0.001). DCA decreased plasma lactate –0.48 (0.82) mmol/L (–20%; *P* = 0.006). Survival of participants was significantly greater than that of a natural history cohort (log-rank *P* = 0.027).

**CONCLUSION:**

Longer-term treatment with DCA, dosed based on *GSTZ1* haplotype, is safe and was associated with a statistically significant improvement in patient motor function, plasma lactate, and survival.

**FUNDING:**

NIH (R01FD005407; R42HD089804), University of Florida Department of Medicine, Saol Therapeutics.

## Abstract 2

In a phase 3 trial, we found dichloroacetate (DCA) was safe and significantly improved morbidity and survival in children with pyruvate dehydrogenase complex deficiency.

## Introduction

Pyruvate dehydrogenase complex deficiency (PDCD) is a rare, primary mitochondrial disease caused by loss-of-function variants in any one of the 5 nuclear genes encoding protein components of the pyruvate dehydrogenase complex (PDC): *PDHA1*, *PDHB*, *DLAT*, *DLD*, and *PDHX* (1, 2). Based on the estimated disease incidence of approximately 1 in 40,000 live births in the United States, the estimated prevalence of PDCD exceeds 500 individuals (3). PDC is located in the mitochondrial matrix, enzymatically catalyzes the decarboxylation of glucose-derived pyruvate to acetyl coenzyme A, and is responsible for a key step in generating ATP (4). PDC is inhibited by tissue-specific isoforms of pyruvate dehydrogenase kinase that reversibly phosphorylate the E1α subunit of PDC (4–6).

PDCD is primarily a disorder of mitochondrial energy failure that particularly affects tissues dependent on high rates of ATP production; thus, the primary manifestations are progressive neurological and neuromuscular degeneration and early death (2). PDCD is frequently associated with elevated circulating levels of plasma lactate and lactic acidemia, which can be fatal (2, 7). No FDA-approved pharmacological treatments for PDCD exist, and management has relied on general supportive care; nutritional supplements of various vitamins, cofactors, and antioxidants; and ketogenic diets (8–10).

Dichloroacetate (DCA) is an orally administered structural analog of pyruvate, an endogenous pyruvate dehydrogenase kinase inhibitor. It acts by inhibiting the phosphorylation and inactivation of the PDC (11–14) and by stabilizing the E1α subunit of the enzyme upon repeated exposure to the drug (15–17). Initial anecdotal reports suggest efficacy of DCA in other causes of congenital lactic acidosis (CLA) (18). A subsequent trial in a heterogeneous population with CLA showed a reduction of basal and postprandial increases in plasma and cerebrospinal fluid lactate but no effect on neurological or other clinical outcome measures (19). However, DCA appeared to increase survival in those with PDCD relative to participants with CLA due to respiratory chain disfunction (20).

In some studies of DCA, treatment has been limited by the development of reversible peripheral neuropathy (21). In 2012, it was found that different haplotypes of *GSTZ1* encoding glutathione transferase (of which the zeta1 family isoform encodes maleylacetoacetate isomerase, an enzyme of tyrosine catabolism; the gene was previously named *MAAI*) caused variable plasma DCA clearance (22). Across multiple ethnic groups, the EGT haplotype of *GSTZ1* is the most common (45%–55%), and individuals possessing at least 1 *EGT* allele metabolize DCA faster than people lacking this haplotype (22). In contrast, the KRT haplotype of *GSTZ1* is among the least frequent (<10%) in many populations and causes a much slower rate of DCA metabolism (22). Thus, *GSTZ1*, the gene encoding the primary enzyme involved in DCA metabolism, influences the toxicokinetics of DCA (22, 23). A technique for prospectively identifying participants at risk for high exposure to DCA was subsequently developed to guide dosing (24).

This phase III trial evaluated the efficacy and safety of *GSTZ1* haplotype–guided dosing of DCA treatment in participants with PDCD. The trial specifically compared the effects of DCA with a placebo on motor function and plasma lactate levels. (ClinicalTrials.gov NCT02616484). We also report initial findings of a separate protocol that compared survival in participants of this study with that of a published natural history cohort, matched by age and sex.

## Results

### Participant characteristics.

**Figure 1:**
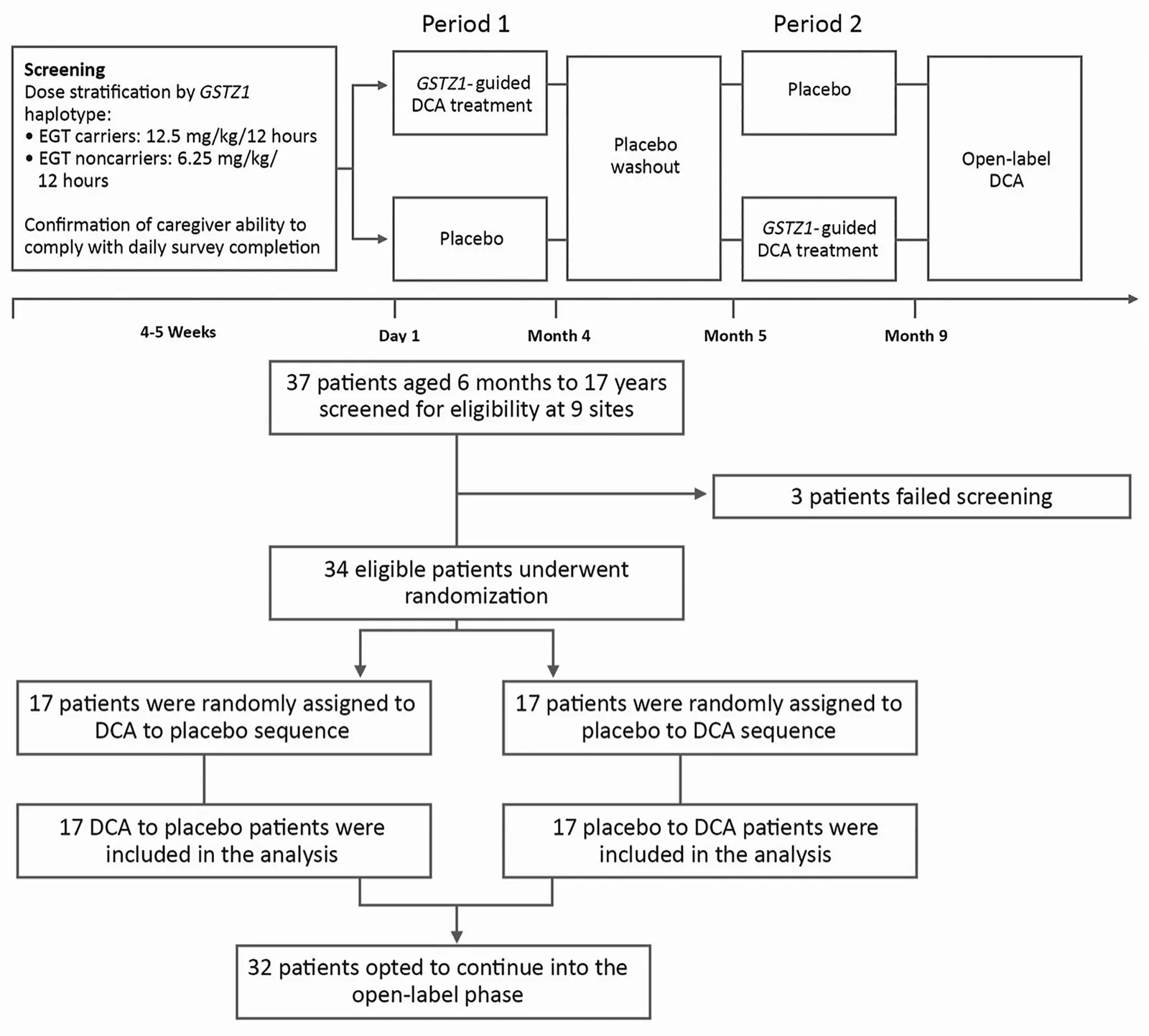
Trial design and participant disposition. The overall design of the trial, in which participants with PDCD were randomly allocated to 1 of 2 treatment sequences with *GSTZ1* haplotype–guided dosing. Each participant received each treatment for 4 months. During the screening period, the diagnosis of PDCD due to known pathological variants was confirmed by molecular testing. All participants underwent *GSTZ1* haplotype analysis for dose stratification. Parents/caregivers completed daily ObsRO surveys to demonstrate greater than 80% compliance for each parent/caregiver with the requirement for daily survey completion during the trial. DCA was supplied as a 50 mg/mL solution for oral administration. Dosages were stratified according to *GSTZ1* haplotype: fast metabolizers (*EGT* carriers) received 12.5 mg/kg/12 hours and slow metabolizers (*EGT* noncarriers) received 6.25 mg/kg/12 hours. The dose could be altered to accommodate weight changes during the study. After the second treatment period, participants could enter an open-label phase, during which all participants received DCA with adjustments for weight changes. Parents/caregivers were given the option to complete the ObsRO survey daily for the first 30 days of the open-label phase and were requested to complete the survey on each of the 7 days before each open-label clinic visit.

Thirty-seven participants were screened, of whom 3 (8.1%) failed screening (1 declined to participate and the families of 2 participants did not meet compliance requirements) and 34 were randomly allocated to treatment (Figure 1). All 34 participants completed the double-blind portion of the study and were included in the analysis, and 32 participants continued treatment in the open-label portion. Baseline characteristics were well-balanced between the 2 sequences (Table 1).

As of the July 17, 2024, data cutoff, all 32 participants who entered the open-label phase of the study had completed at least 1 open-label visit (≥6 months). Twenty-nine participants remained on open-label treatment. The maximum exposure to DCA in the open-label phase was 1,111 days. The minimum duration of exposure was 227 days.

### Efficacy.

The parent-driven observer-reported outcomes (ObsRO) daily survey (25) originally contained 11 domains; the outcome measure agreed to with the FDA was adapted for this trial to include the 5 ObsRO domains related to motor function (weakness, incoordination, hypotonia/low muscle tone, rigidity/hypertonia, and involuntary movements) (ObsRO_motor_) graded on a Likert scale from 0 (mild or absent) to 4 (extremely severe), with the overall score ranging from 0 to 20. ObsRO_motor_ least squares mean (LSM) changes from baseline were –0.49 and –0.79 for DCA and the placebo, respectively (LSM difference, 0.29; 95% CI, –0.60 to 1.19; *P* = 0.512).

An exploratory analysis recommended by the FDA found a floor effect (i.e., a lower limit), below which participants had a limited ability to improve, biasing the group toward worsening. A nonstatistically significant trend toward improvement was observed among participants who could both improve or decline, as defined by a baseline ObsRO_motor_ score of at least 8 (*n* = 15, –1.12; *P* = 0.066). To assess the effect of longer durations of therapy, baseline ObsRO_motor_ scores were compared with the most recent ObsRO_motor_ score observation as of the open-label extension phase data cutoff date (July 17, 2024) (Table 2). Longer-term treatment (double blind plus open label) resulted in a significant treatment effect (*P* = 0.002).

**Figure 2:**
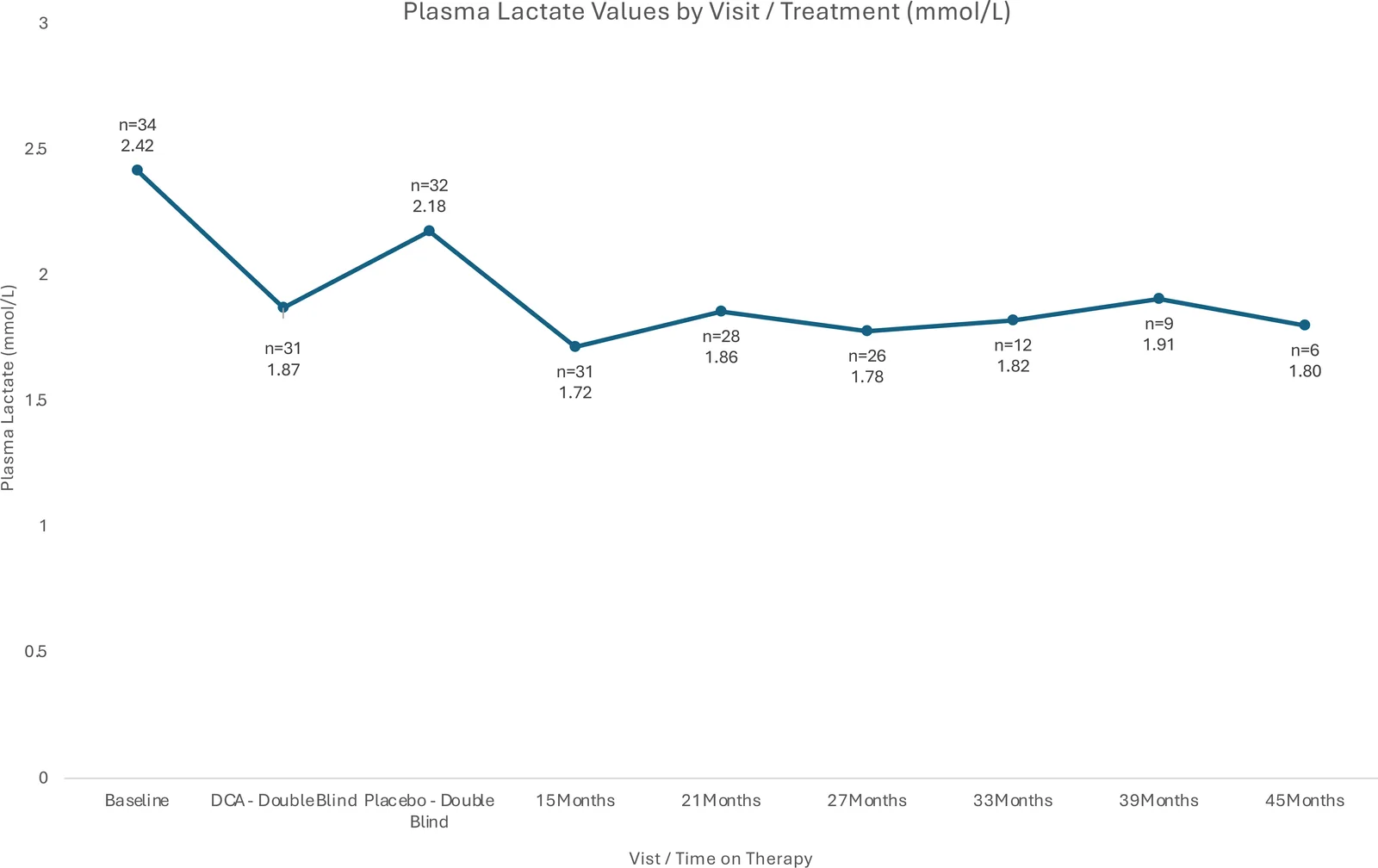
Average plasma lactate values at baseline, end of the placebo and DCA double-blind periods, and during open-label extension. Plasma lactate values are shown (mmol/L).

Plasma lactate levels were evaluated as a secondary endpoint. DCA was associated with an average reduction in plasma lactate of –0.48 (0.82) mmol/L relative to the placebo (*P* = 0.006). The lactate-lowering effect of the drug was sustained throughout the double-blind and open-label periods (Figure 2). During the double-blind portion of the study, lactate levels of more than 2.0 mmol/L (the upper limit of normal) were observed in 11/34 (32%) and 18/34 (53%) of participants receiving DCA or the placebo, respectively. Of the 30 participants with available lactate data, 23 (77%) participants remained within the normal range of less than 2.0 mmol/L during the open-label period, suggesting a durable lactate-lowering benefit associated with long-term therapy.

Five of the 34 randomized participants had seizures during the baseline period. The average (SD) within-individual difference for total seizure episodes, including those with no seizures, was 10.5 (66.7; *P* = 0.364). Several participants experienced a reduction in seizures during the trial. One had complete resolution of seizures while receiving DCA, 2 had clinically meaningful reductions in the average number of seizures, 1 remained stable while receiving DCA, and 1 did not have seizures after the baseline period. Neither electromyography nor nerve conduction velocity measurements were performed on any participant.

There was no statistically significant difference between groups for the average (SD) total number of hospitalizations/emergency department visits (–0.1 [1.14]) or vomiting episodes (4.3 [42.1]).

### Safety.

No patient was metabolically decompensated at entry into the trial. Twenty-five (73.5%) participants reported 76 adverse events during treatment with DCA, and 27 (79.4%) participants reported 73 adverse events during treatment with the placebo (Table 3), including constipation, diarrhea, fatigue, and muscular weakness in the DCA group and cyclic vomiting syndrome, flatulence, and salivary hypersecretion in the placebo group.

During the double-blind portion of the study, there were no events of peripheral neuropathy. During the open-label extension, 1 event of mild neuropathy that was possibly related to therapy occurred after 18 months of treatment in a participant aged 17.5 years. One participant discontinued DCA treatment for personal family reasons after completing the double-blind portion of the study and died of pneumonia caused by SARS-CoV-2 infection 3 months later.

Seventy percent (24 of 34) of participants reported being on a ketogenic diet at baseline. The ketogenic diet ratio (proportion of fat to carbohydrate plus protein calories) mostly ranged from 2.5:1 to 4.1:1 based on individual patient food diaries. The overall change in plasma ketone β-OHB levels was evaluated as part of the research laboratory analysis for monitoring ketosis status, which was undertaken for all participants, regardless of ketogenic diet adherence. Among participants in the intent-to-treat population, individuals receiving DCA had an average (SD) change of β-OHB from baseline of 0.23 (1.40) mmol/L [2.39 mg/dl] vs. –0.20 (0.80) mmol/L [2.08 mg/dl] for participants receiving the placebo. The overall treatment effect average β-OHB difference of 0.44 (0.26) was not statistically significant (*P* = 0.098). In the 24 participants who were adherent to a ketogenic diet (included β-OHB values for baseline and end-of-treatment periods and a baseline β-OHB > 0.3 mmol/L), the average baseline β-OHB was 2.7 mmol/L. After treatment with DCA in the double-blind portion of the study, the average β-OHB level increased nonsignificantly to 3.0 mmol/L, whereas a decrease to 2.3 mmol/L was observed after treatment with the placebo (average difference DCA vs. placebo 0.6 mmol/L). Elevations in β-OHB stabilized in most participants and did not appear to continue to rise on longer-term DCA exposure (>12 months). No events of ketoacidosis were reported.

### Survival.

Of the 34 DCA-treated participants in the trial, 33 (97%) were eligible for this analysis; 1 participant was excluded for having a non-*PDHA1* pathogenic variant. After exclusion of those who had previously received DCA (*n* = 6) and those whose last known age was less than or equal to the minimum age at DCA initiation in the DCA treatment cohort (*n* = 13), 34 participants were identified from the natural history cohort from DeBrosse et al. (26). Comparisons of the pathological variants between the DCA-treatment participants in this study and the untreated natural history cohort were similar (Table 4). The degree of mutational concordance between the 2 patient cohorts — this study and the natural history cohort — were also similar (Table 5). Using sex and age at PDCD onset, plus assigning a matched index date, 28 DCA-treated participants were 1:1 matched to untreated participants. The average age (SD) at the index date was 5.1 (± 5.1) years, and 71.4% were female.

**Figure 3:**
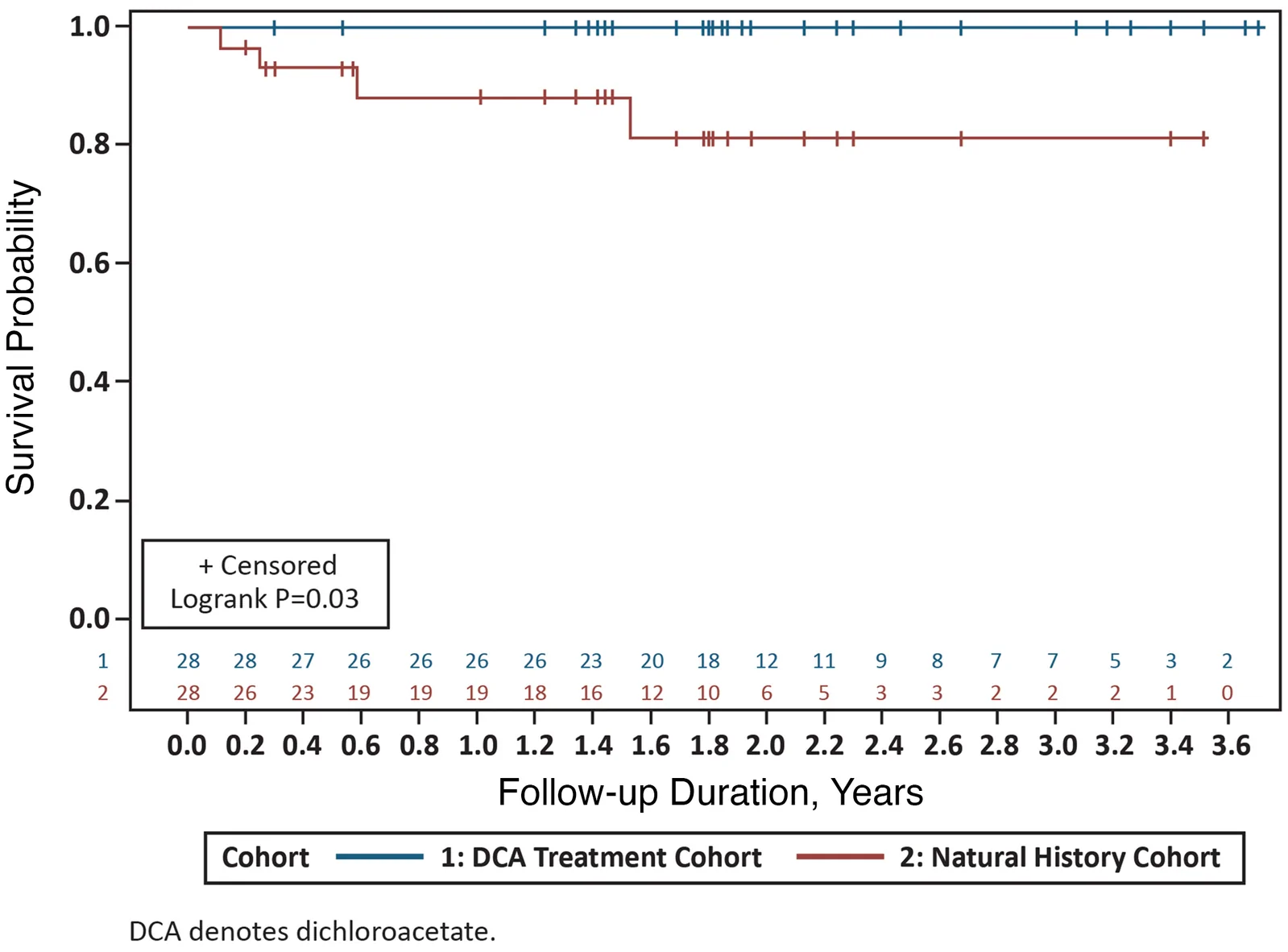
Overall survival in the sex- and age-matched groups with number of participants at risk. Overall survival was assessed using Kaplan-Meier curves and a log-rank test.

When comparing survival of DCA-treated participants with the untreated natural history cohort, differences in age at PDCD onset after imputation remained between the DCA-treated and untreated groups (average age, 0.4 vs. 1.9 years). Furthermore, differences were seen in the implementation of a ketogenic diet (64.3% vs. 71.4%, standardized difference [SDiff] = 0.15), as well as in the use of vitamins and supplements as background therapies: thiamine was taken by 64.3% and 75.0% (SDiff = 0.24) and l-carnitine by 32.1% and 53.6% (SDiff = 0.44) in the DCA-treated and untreated groups, respectively (Table 6). Levetiracetam, the most commonly used antiseizure medication, was administered to 17.9% of participants in the treatment group, whereas phenobarbital and topiramate were the most frequently used antiseizure medications in the untreated group (21.4% each; Table 6). Hypotonia (96.4% vs. 78.6%; SDiff = 0.56) and developmental delay/intellectual disability (78.6% vs. 89.3%; SDiff = 0.30) were the most common signs and symptoms of PDCD in the treated and untreated groups (Table 6). Seizures were reported for 32.1% of the DCA-treated and 53.6% of the untreated participants (SDiff = 0.44) (Table 6). Across 39.9 person-years in the untreated natural history cohort, 4 deaths occurred, for an incidence rate (95% CI) of 10.02 (2.73–25.65) deaths per 100 person-years. In the DCA treatment group, 59.3 person-years of follow-up were included, and no deaths of participants remaining on DCA had occurred as of July 24, 2024. The resulting incidence rate (95% CI) was 0.00 (0.00–5.05) deaths per 100 person-years (*P* = 0.027) (Figure 3). As reported, 1 patient in our cohort had PDCD due to a homologous pathological variant affecting *PDHX* and was therefore excluded from the survival analysis. However, the participant with the *PDHX* variant was included in all of the other phase III clinical trial analyses.

## Discussion

This study evaluated the efficacy of DCA in children with PDCD, for whom no FDA-approved pharmacological treatments exist. The present clinical trial differs from previous evaluations of DCA in PDCD (19, 20) in that DCA dosage was tailored according to participant *GSTZ1* haplotype. Previous studies showed that participants with at least 1 *EGT* haplotype (*EGT* carriers) metabolized DCA more quickly than *EGT* noncarriers (22). A rapid genotyping/haplotyping assay for pharmacogenomic tailoring of DCA dosing was subsequently developed to mitigate or prevent adverse events in participants receiving long-term treatment (24). Although *EGT* carrier status is not known to affect the PDCD phenotype, there is no plausible reason to assume that it does.

When this study was initially conceived, there were no validated biochemical biomarkers or clinical assessment tools applicable as primary outcome measures for a randomized controlled trial in PDCD. Consequently, based in part on the advice and support of the FDA and parents of affected children, we generated a potentially novel ObsRO_motor_ daily survey (25) employed here as an objective, user-friendly means of prospectively assessing patient functionality in a home environment. Patient muscle tone and function were considered by caregivers to be a particularly critical indictor of a child’s clinical status, which was therefore employed as a primary outcome variable. Accordingly, the ObsRO_motor_ daily survey (25) was used by parents/caregivers to capture information across 5 motor domain elements to assess the participant’s daily functionality. This endpoint did not demonstrate statistical significance. In retrospect, we recognize that ObsRO_motor_ improvement may be difficult to achieve for multiple reasons: PDCD is associated with many structural neurological abnormalities that cannot be reversed and that drive much of the neurological deficit; there is considerable heterogeneity in the neurological deficits with PDCD; the disease is progressive; the sample size is small due to the rarity of the disease; the disease had no previously validated measurement tools; and participants who had low baseline values (<8) on the ObsRO_motor_ had a lower disease burden, and therefore less room to demonstrate improvement, exhibiting a floor effect. In this regard, it is noteworthy that the ObsRO tool was developed from what caregivers reported they observed when their child was acutely ill and metabolically decompensated (25). In contrast, the patients enrolled in this trial were chronically ill but metabolically stable. Thus, it would be expected that application of the ObsRO survey would exhibit a floor effect and be most applicable to those children enrolled in the study who exhibited severe muscle weakness at baseline. Accordingly, due to the patients’ clinical status upon entry, the overall baseline ObsRO_motor_ average was lower than expected, given the disease severity. After examining the distribution of baseline ObsRO_motor_ scores, we found 19 of 34 participants (56%) had limited motor function disease severity as reported by their caregivers, and there was no prespecified method to control for interrater variability or to align the caregiver and physician on the proper characterization of the participant. As a result, many participants had a limited ability to demonstrate significant improvement, as measured by the instrument used in the study.

Although the overall study cohort did not meet the prespecified primary motor endpoint, clinical improvements were observed in participants with higher baseline ObsRO_motor_ scores (i.e., more severe motor function impairment). Using the last reported ObsRO_motor_ measurement versus baseline in the ongoing open-label phase of the study, both the full intent-to-treat population and high baseline (≥8) participants exhibited clinically meaningful improvement (>20%) and statistically significant reductions in ObsRO_motor_. These data suggest that longer DCA exposure yields greater treatment benefits. Even participants having a low baseline score (<8) exhibited modest improvement or disease stability, a notable achievement in a progressive neurodegenerative disorder.

Average baseline plasma lactate levels were only modestly elevated across the cohort, which is consistent with study exclusion criteria prohibiting enrollment of any children with acute lactic acidosis and metabolic instability. Nevertheless, a significant and sustained reduction in plasma lactate levels was observed in participants treated with DCA (Figure 2). No cases of lactic acidemia were recorded, indicating that no metabolic decompensation was observed. Hyperlactatemia and lactic acidosis are common metabolic complications of PDCD (1, 2, 26–28). Individuals with inborn errors of mitochondrial metabolism are at increased risk of immune dysfunction and infection (29, 30), and lactate is known to be a significant immunosuppressant (31). Thus, a reasonable postulate is that the chronic lactate-lowering effect of DCA may have immune-modulating effects beneficial to the host. Moreover, PDC resides in both the mitochondrial matrix and the nucleus of cells, where it interacts with the epigenome via acetylation and lactylation of proteins (23, 32). In addition to reducing circulating lactate, DCA also may decrease lactylation of proteins, which is a process that is also associated with negative outcomes in participants with cancer and other inflammatory diseases (31, 33, 34). In the future, monitoring lactylated amino acids may be a worthwhile objective as a biomarker for PDCD.

DCA readily crosses the blood-brain barrier in rats, activating the PDC and lowering brain lactate (35–38). Oral DCA also lowers cerebrospinal fluid lactate in children with PDCD or mitochondrial respiratory chain diseases (19). Recent evidence suggests the drug also reduces the phosphorylated proportion of PDC in tumor tissue of adults with recurrent glioblastoma, resulting in reversal of aerobic glycolysis (Warburg metabolism) in the tumors (39). Although we lack direct evidence to support this notion, it is highly plausible that oral DCA enters the CNS of PDCD study participants, with potential implications for improved CNS function.

In this study, nonsignificant β-OHB increases of up to 3 mmol/L were observed in some participants who adhered to a ketogenic diet during DCA treatment. These elevations stabilized in most cases and did not continue to rise with longer-term DCA exposure (>12 months); there were no reported adverse events of ketoacidosis. It is known that stimulation of PDC activity increases β-OHB concentrations (40). Thus, DCA, by increasing conversion of pyruvate to acetyl CoA, would lead to increased β-OHB production. Monitoring of β-OHB levels is recommended for the treatment of patients with PDCD on a ketogenic diet, with a target goal of β-OHB maintenance of about 3.0 to 5.0 mmol/L (9). Thus, it is plausible the addition of DCA may allow patients with PDCD whose nutritional intake is high in fats on a ketogenic diet to be transitioned to a lower ratio diet and consume a greater percentage of their dietary calories as carbohydrates and protein.

*GSTZ1* haplotype–guided DCA dosing in this study resulted in adverse events similar to the placebo. No cases of peripheral neuropathy were reported during the double-blind period; however, during the open-label period, 1 participant developed mild peripheral neuropathy after 18 months of treatment and later withdrew after relocation. This was an unusual presentation as it occurred after 18 months of exposure and was therefore not likely due to the more rapid onset of neuropathic events reported in the literature, but could possibly reflect chronic exposure to the drug (21). Peripheral neuropathy is also a potential complication of PDCD (1, 2) and may have been reflected as such in this participant.

We acknowledge that small differences in disease severity could influence survival outcome when relying on small historical control groups. Nevertheless, treatment with DCA was associated with a statistically significant difference in survival compared with an untreated natural history cohort, with an overall estimated reduction in the risk of death ranging from 97% to 99%. All of the DCA-treated patients with PDCD survived, that is, DCA treatment was associated with a reduction in mortality from 4 individuals to 0. Importantly, all patients with PDCD in both the natural history and current study received the same standard of care with the exception of DCA. These findings provide further evidence that similar participants with PDCD receiving DCA for the same range of years lived longer than individuals who did not receive DCA. This conclusion is consistent with a previously reported randomized controlled trial of oral DCA in 43 children with various causes of CLA, 10 of whom had PDCD (19). In a later publication (20), survival of this cohort of patients was compared between those who had PDCD (*n* = 10) and those who had mitochondrial respiratory chain functional impairment (*n* = 26). Although not statistically significant due to the small sample size, patients with PDCD treated with DCA appeared to have longer lifespans when compared with patients with other causes of CLA.

Limitations exist that are inherent to external control arm studies in rare diseases, especially those using a historical control group. To the extent possible, the limitations were addressed in this study through exact matching on sex and age at the start of the analysis, as well as varying the assumptions used for imputing age at onset and conducting the matching with the natural history untreated PDCD population.

### Perspective.

The results of this phase III clinical trial of DCA in PDCD demonstrate the following: (a) Chronic oral DCA is well-tolerated and safe for at least a duration approaching 4 years, based on this study and others (19, 41) in children with PDCD or other mitochondrial disorders. (b) DCA causes a statistically significant and sustained reduction in plasma lactate levels. This drug-induced reduction in plasma lactate to or near normal levels, together with a decrease in the frequency of metabolic decompensation among treated patients with PDCD and a growing clinical literature relating even mild hyper-lactatemia to serious illnesses, speaks to the importance of maintaining reduced circulating lactate concentrations in chronically ill participants, such as those with PDCD. (c) Despite the inherent limitations in comparing our patients’ survival to that of historical controls, the study group was well-matched to a natural history control cohort, demonstrating a beneficial effect of chronic DCA on survival in PDCD. (d) Together, these results have led to a new drug application by Saol Therapeutics to request FDA approval of DCA as the first pharmacotherapy for this devastating pediatric disease.

### Conclusion.

*GSTZ1* haplotype–guided dosing resulted in adverse events similar to the placebo, with only 1 case of peripheral neuropathy that occurred 18 months after exposure during the open-label extension period, a substantial improvement over prior studies done without *GSTZ1* haplotype–guided dosing. Treatment with DCA showed a statistically significant survival difference compared with an untreated natural history cohort. Further, with longer-term treatment, statistically significant differences in motor function were seen. Lastly, lactate levels were lower during DCA treatment and sustained during the open-label extension period.

## Methods

### Sex as a biological variable.

*PDHA1*-related PDCD is an X-linked disease, and males are generally considered to be more severely affected than females by virtue of random X inactivation. Sex was not considered as a biological variable in this study, but the findings were expected to apply to both sexes.

### Participants.

From June 2020 through August 2023, we screened and enrolled participants at 9 sites in the United States (ClinicalTrials.gov NCT02616484). Participants aged 6 months through 17 years were eligible if they had the characteristic clinical or metabolic features of PDCD; measurable signs/symptoms on the ObsRO scale (25); and a known pathogenic variant in a primary specific PDC gene, *PDHA1*, *PDHB*, *DLAT*, and *PDHX*. Biochemical confirmation of PDC enzymatic deficiency was required for novel PDC gene variants not previously described. Of the 34 participants randomly allocated to treatment, 33 had pathogenic variants in *PDHA1* and 1 in *PDHX*. All participants were allowed to remain on baseline medications, and no attempt was made to adjust these medications or dietary interventions, such as a ketogenic diet, provided they were well-tolerated by participants.

### Trial design and treatment.

Participants were randomly assigned via a web-based system to two 4-month duration treatment sequence groups: DCA, 1-month washout, placebo; or placebo, 1-month washout, DCA (Figure 1).

### Endpoints and assessments.

The primary endpoint was the average change from baseline in the participant’s daily total score on the ObsRO_motor_ over the last 7 days of the 4-month treatment period in the DCA treatment period minus the average change from baseline in daily total score in the placebo treatment period.

The frequency of seizures was documented throughout the double-blind and open-label periods of the trial, but neither formal electromyography nor nerve conduction velocity testing was performed. The types of antiseizure medications for the DCA-treated and untreated groups are listed in Table 6. Secondary endpoints included change in plasma lactate levels for DCA- and placebo-treated participants at baseline and visit 4 (approximately 4 months after baseline) and visit 6 (approximately 9 months after baseline) and the numbers of seizures, vomiting episodes, and hospitalizations/emergency department visits during the treatment periods.

In a separate protocol, we compared survival differences between children diagnosed with PDCD and treated with DCA in the present study with participants in a published untreated natural history cohort (26). Exclusion criteria for participants from the natural history cohort were (a) no demonstrated evidence for a pathogenic or likely pathogenic variant in *PDHA1*; (b) receipt of DCA at any time; and (c) a last known age less than or equal to the minimum age at DCA initiation in the DCA treatment group (0.9 years). The index date for DCA-treated participants was defined as the age at DCA initiation in the present study; each comparator participant was assigned an index date of the same age as the matched treated participant. For the assessment of survival, participants were followed using an intent-to-treat approach from the index date until death, or for those with no record of death, until the last age at which the participant was confirmed to be alive. The primary outcome was overall survival, defined as the time in months from the index date until death from any cause.

### Statistics.

The primary endpoint of this trial was the average change from baseline in the participant’s daily ObsRO_motor_ score over the last 7 days of the 4-month treatment period in the DCA-treated period minus the average change from baseline in the daily total motor score for the last 7 days of the 4 months in the placebo-treated period. We assumed that if the distribution was approximately normal and there was a 75% probability that the average ObsRO_motor_ score for the placebo period would be greater than that of the DCA period, then the study would need 22 participants (11 per treatment sequence) to have 80% power at a 0.05 significance level using a paired, 2-tailed *t* test or Wilcoxon’s test. Assuming a 20% dropout rate, it was estimated that 30 participants (15 per group) would be needed to obtain 12 evaluable participants per treatment sequence. The LSM was reported rather than the observed average because the change from baseline was the dependent variable, and the comparison was based on a mixed model for repeated measures (MMRM). The LSM is adjusted by other specified effects in the model, as described below.

The treatment effect was compared between the DCA-treated and placebo-treated participants using the MMRM, with treatment, sequence, and period as a fixed effect and participant within sequence as a random effect. The treatment effect was also assessed for the 2 ObsRO_motor_ subgroups, which were identified using a threshold baseline total motor score of 8 (i.e., baseline total motor score ≥8 vs. <8). In addition, the treatment effect was assessed for period 1 data only using a paired 2-tailed *t* test.

Changes in plasma lactate and β-OHB levels were compared using a paired 2-tailed *t* test and an MMRM model. The total number of seizures, vomiting episodes, and hospitalizations/emergency department visits were compared using an MMRM model and a paired 2-tailed *t* test. All analyses were conducted using SAS version 9.4 (SAS Institute, Inc.).

We compared overall survival among the DCA-treated participants with an external natural history cohort of untreated participants with PDCD. Comparator participants were matched 1:1 with the DCA-treated participants based on sex and age at PDCD onset. Overall survival was assessed using Kaplan-Meier curves and a log-rank test. For analyses that included no deaths in the DCA-treated group, the relative risk of death for the DCA-treated participants versus the historical control group was estimated using a logistic regression model with time as an offset; analyses that included 1 death in the DCA-treated group used a Cox proportional hazards model. A *P* value less than 0.5 was considered significant.

### Study approval.

The trial was approved by the IRB at each study site and conducted under an investigational new drug license (IND 028625) issued by the FDA. The study was conducted in accordance with the US Code of Federal Regulations (CFR), 21 CFR 50; the Good Clinical Practice guidelines; and the ethical principles outlined in the Declaration of Helsinki. A data and safety monitoring board reviewed all serious adverse events reported throughout the study and reported their conclusions to the study’s principal investigator and the relevant site-specific investigator and IRB. Data and safety monitoring board reports were provided to the FDA division that oversaw performance under IND 028625. All caregivers provided written informed consent.

### Data availability.

Providing supporting data points for the graphs and means contained in this manuscript could inadvertently jeopardize protected health information in this small (*n* = 34 participants) rare disease trial. Interested researchers in need of additional information should contact SH.

Please email shamm@saolrx.com for access to clinical trial data.

## Author contributions

PWS designed the study. JEA, JKB, LB, GME, MJF, RG, KG, AG, NL, RN, RPS, FS, and JV conducted the research. JKB directed the diagnostic core. CG directed the pharmacology core. SH provided DCA and placebo formulations. EH provided biostatistical support. SHS directed the neurology core. REW conducted the *GSTZ1* genotyping of patients.

## Conflict of interest

SH is employed by Saol Therapeutics, which is the current sponsor of the phase III trial and holds IND 028625. REW is the owner and manager of Medosome Biotec LLC and Praesidio Pharma LLC, both of which may receive royalties from Saol Therapeutics from commercialization and sales of the study drug. MJF has served as a paid consultant and principal investigator on sponsored research agreements with Saol Therapeutics.

## Funding support

This work is the result of NIH funding, in whole or in part, and is subject to the NIH Public Access Policy. Through acceptance of this federal funding, the NIH has been given the right to make the work publicly available in PubMed Central.

- NIH grants (R01FD005407; R42HD089804).
- University of Florida Department of Medicine.
- Saol Therapeutics.

## Supplementary Material

Supplemental data

ICMJE disclosure forms

Supporting data values

## Figures and Tables

**Table 1:**
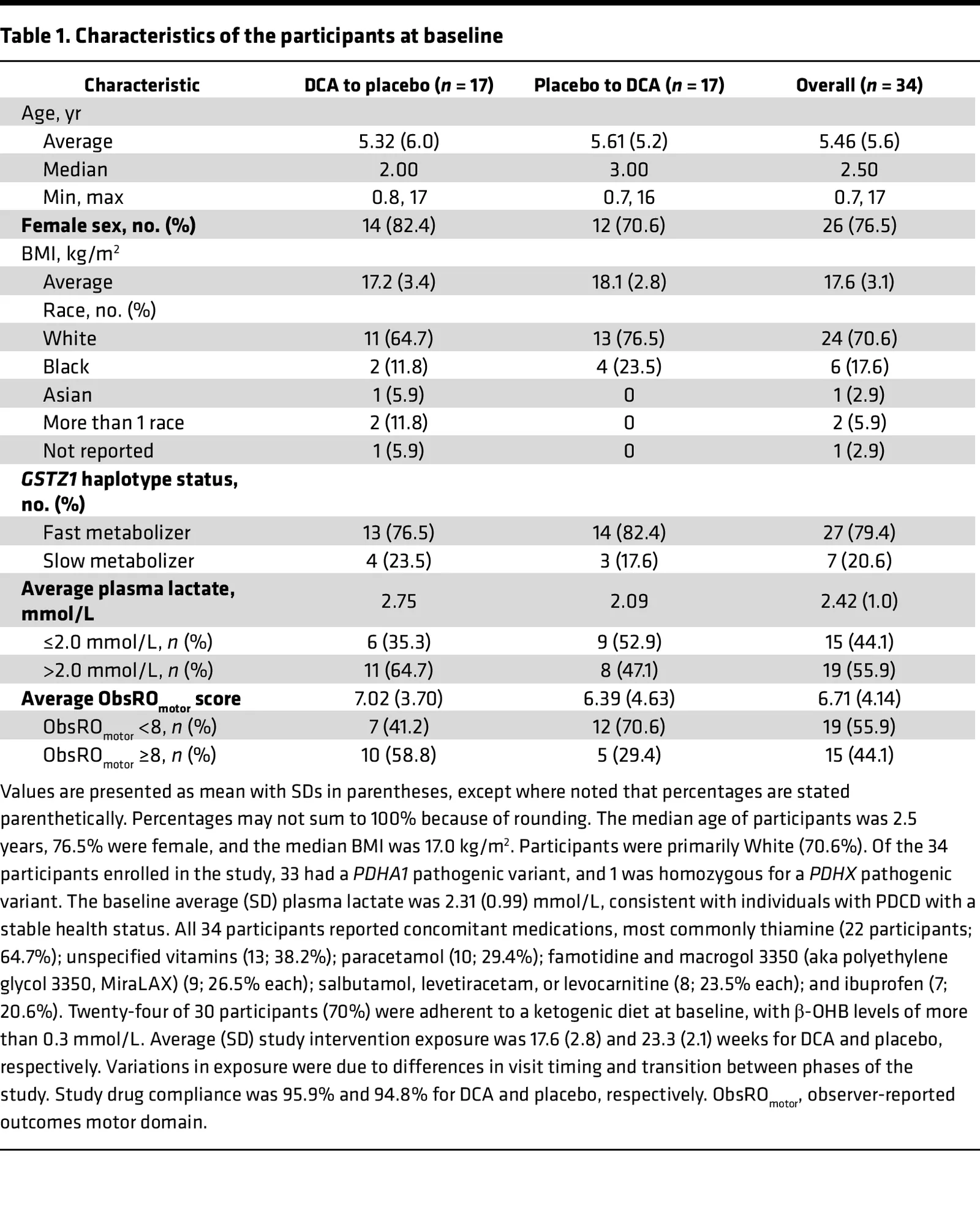
Characteristics of the participants at baseline

**Table 2:**
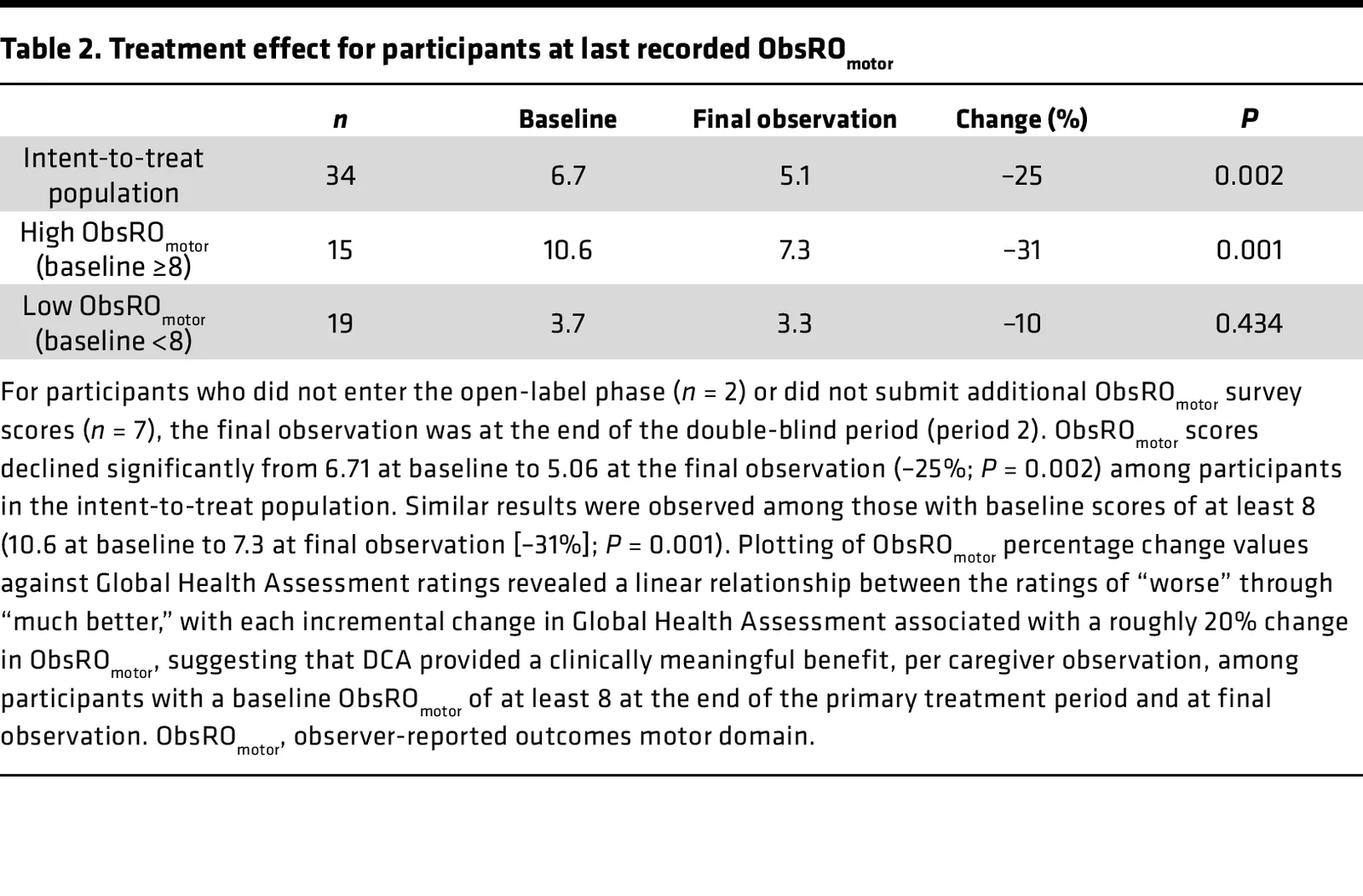
Treatment effect for participants at last recorded ObsRO_motor_

**Table 3:**
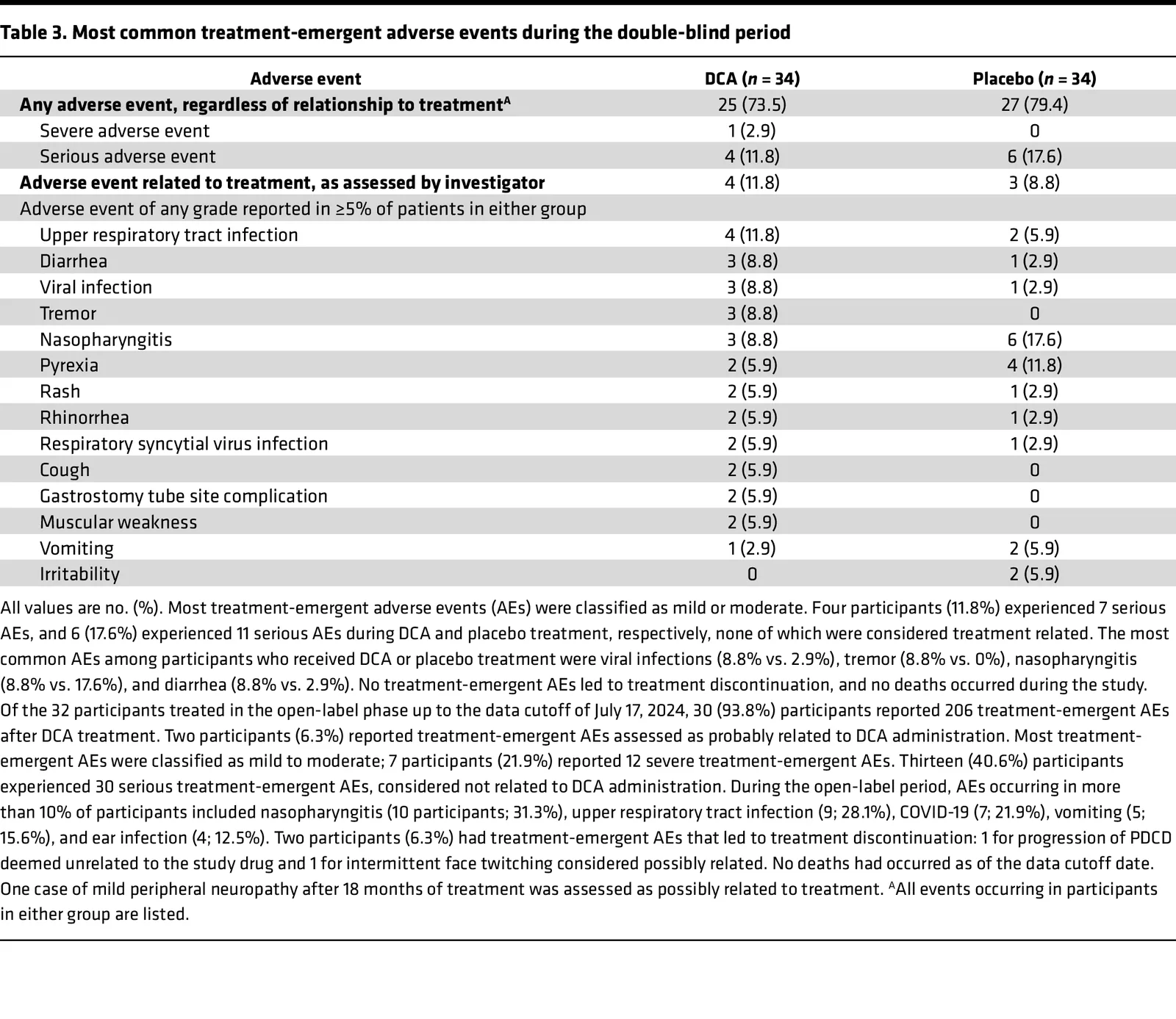
Most common treatment-emergent adverse events during the double-blind period

**Table 4:**
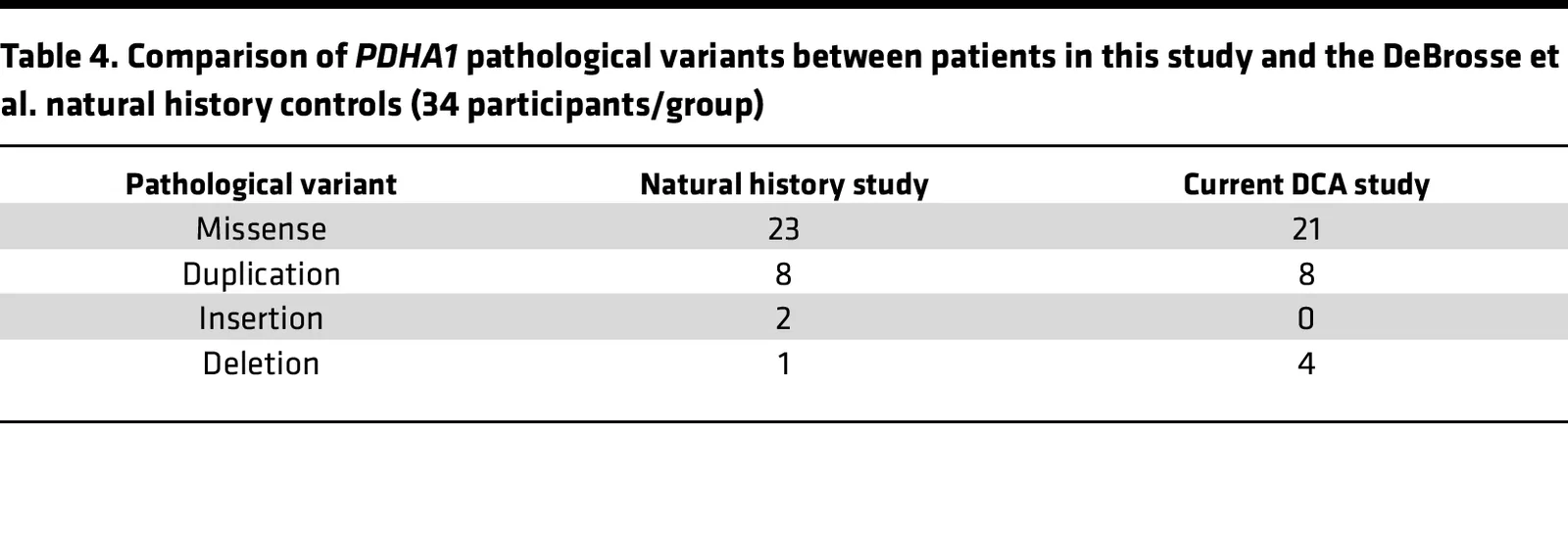
Comparison of *PDHA1* pathological variants between patients in this study and the DeBrosse et al. natural history controls (34 participants/group)

**Table 5:**
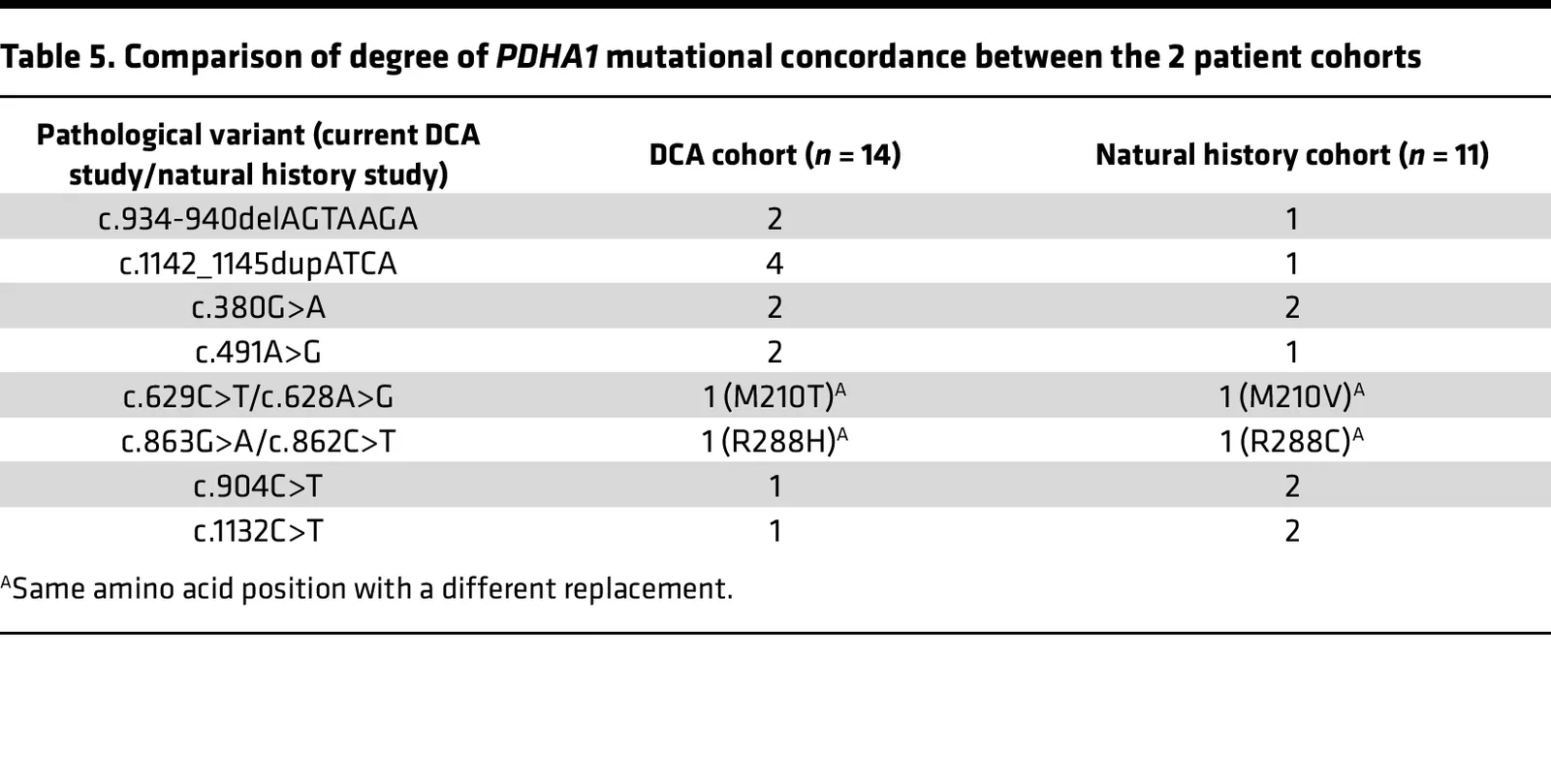
Comparison of degree of *PDHA1* mutational concordance between the 2 patient cohorts

**Table 6:**
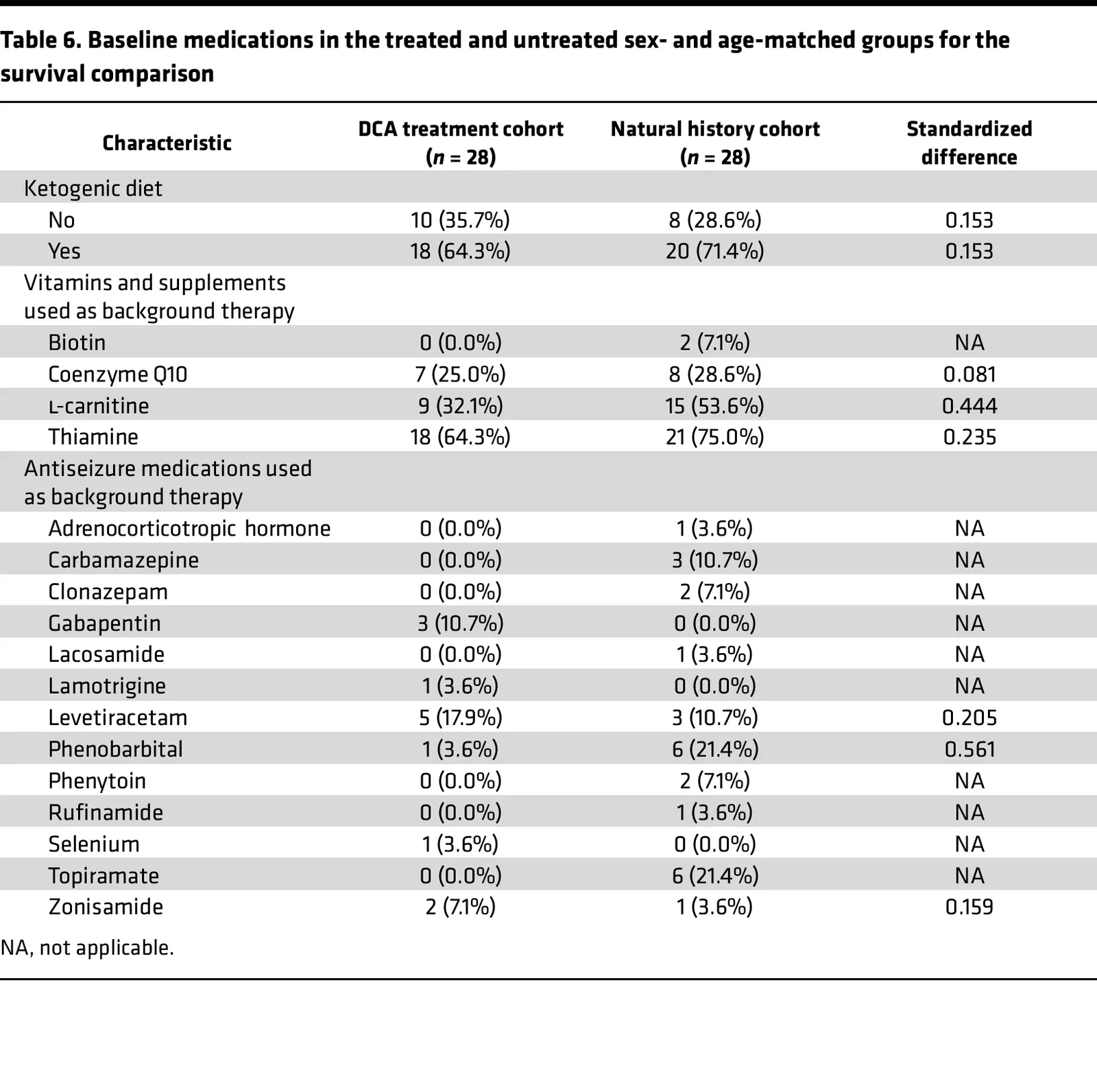
Baseline medications in the treated and untreated sex- and age-matched groups for the survival comparison

## Version 1 09/22/2026

Electronic publication
