# Supplementary material for: Phase III trial of sodium dichloroacetate for pyruvate dehydrogenase complex deficiency in children
Source: JCI Insight. 2026 Sep 22;11(18):e200149. doi: 10.1172/jci.insight.200149 (PMC13596729; doi:10.1172/jci.insight.200149)
Supplement: Supplementary file 1 [file jciinsight-11-200149-s135.pdf]

Supplementary Appendix to:

Phase 3 Trial of Sodium Dichloroacetate for Pyruvate Dehydrogenase Complex Deficiency in Children

Survival Analysis

Figure S1. Sensitivity Analysis: Overall Survival in the Sex- and Age-matched Groups With Extended Follow-up Time

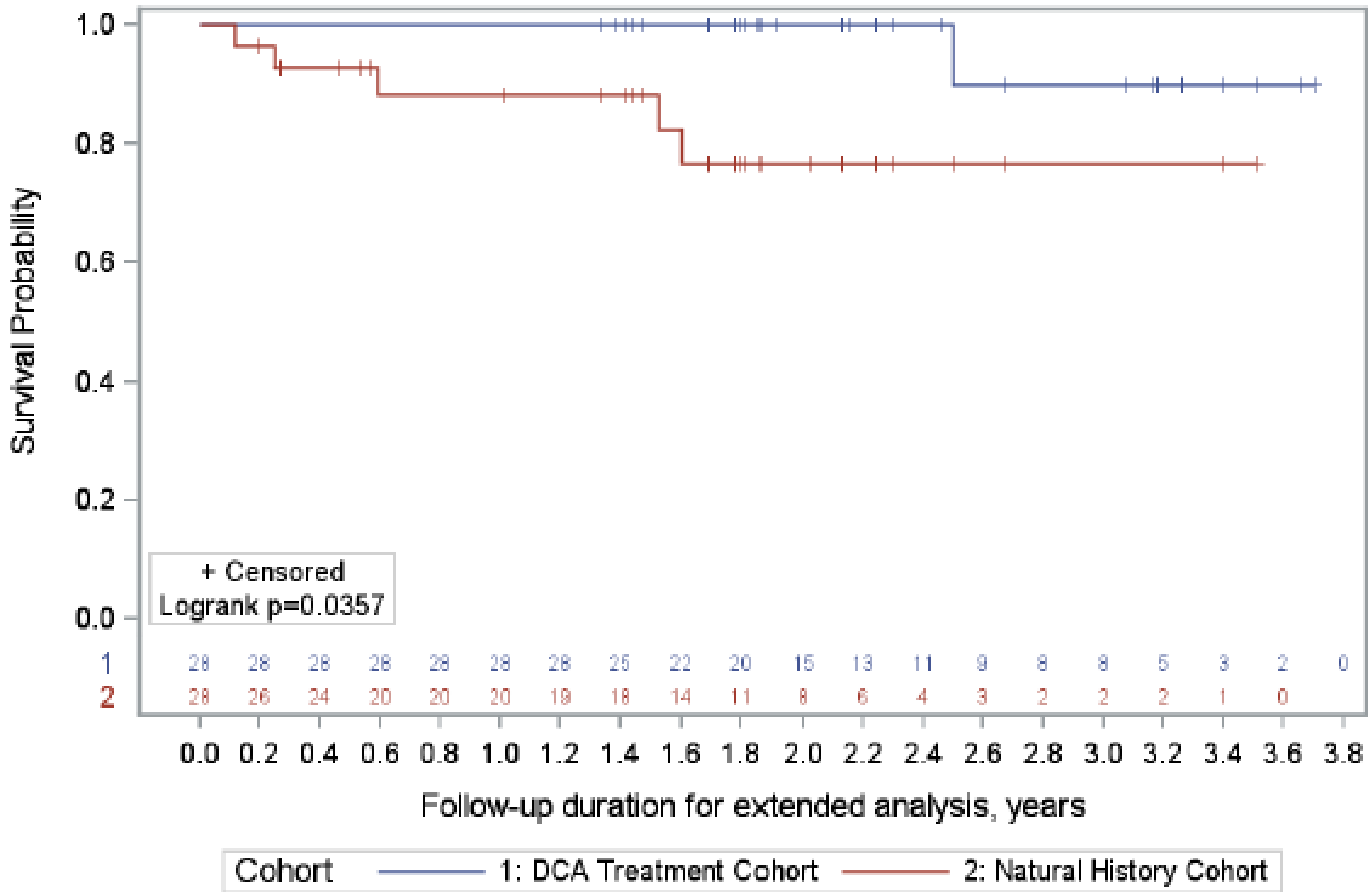

	DCA Treatment Cohort (N=28)	Natural History Cohort (N=28)
**Incidence Rate**		
Number of patients who died, n (%)	1 (3.6%)	5 (17.9%)
Total person-time in cohort, years	64.48	42.51
Events per 100 person-years (95% CI)	1.55 (0.04 - 8.64)	11.76 (3.82 - 27.45)

Note: Extended follow-up time includes post-study discontinuation for the DCA treatment group

Figure S2. Sensitivity Analysis: Overall Survival in the Sex- and Age-matched Groups, Excluding Patients Born in the 1980s

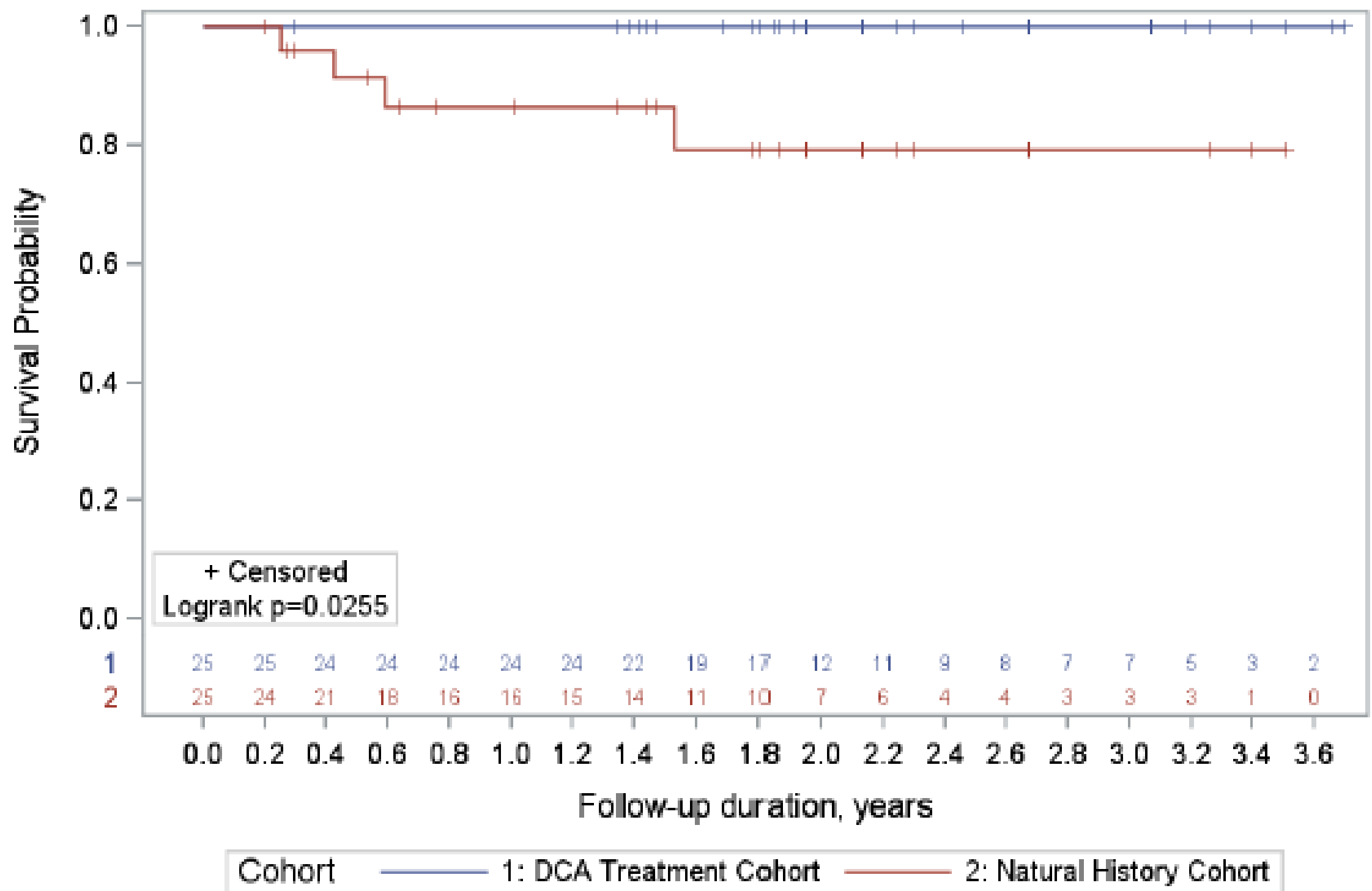

	DCA Treatment Cohort (N=25)	Natural History Cohort (N=25)
**Incidence Rate**		
Number of patients who died, n (%)	0 (0.0%)	4 (16.0%)
Total person-time in cohort, years	55.77	37.66
Events per 100 person-years (95% CI)	0.00 (0.00 - 5.37)	10.62 (2.89 - 27.20)

Figure S3. Sensitivity Analysis: Overall Survival in the Sex- and Age-matched Groups With Extended Follow-up Time, Excluding Patients Born in the 1980s

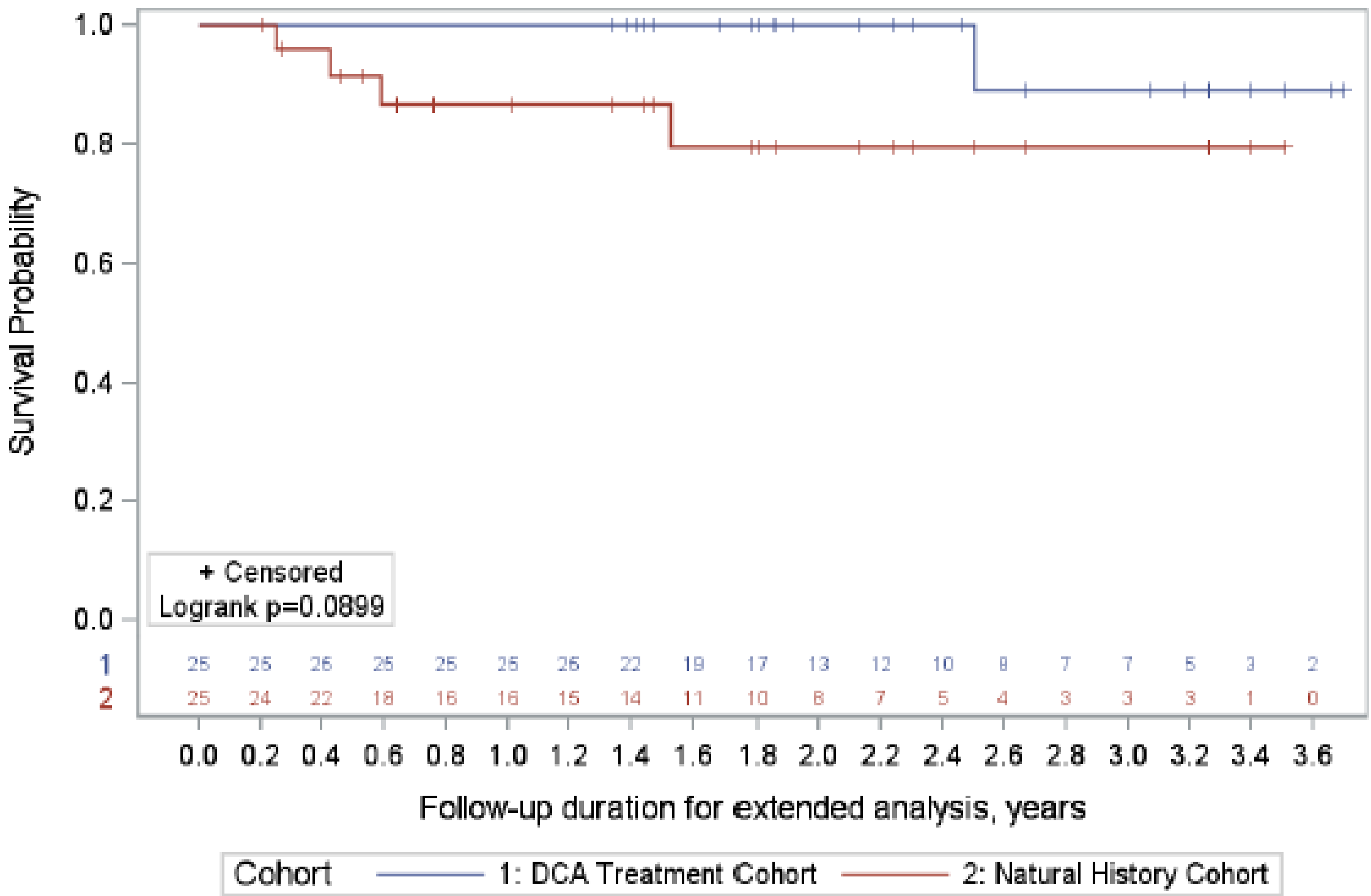

	DCA Treatment Cohort (N=25)	Natural History Cohort (N=25)
**Incidence Rate**		
Number of patients who died, n (%)	1 (4.0%)	4 (16.0%)
Total person-time in cohort, years	57.36	38.37
Events per 100 person-years (95% CI)	1.74 (0.04 - 9.71)	10.43 (2.84 - 26.69)

Extended follow-up time includes post-study discontinuation for the DCA treatment group

Figure S4. Overall Survival in the Age-matched Groups With Number and Subjects at Risk

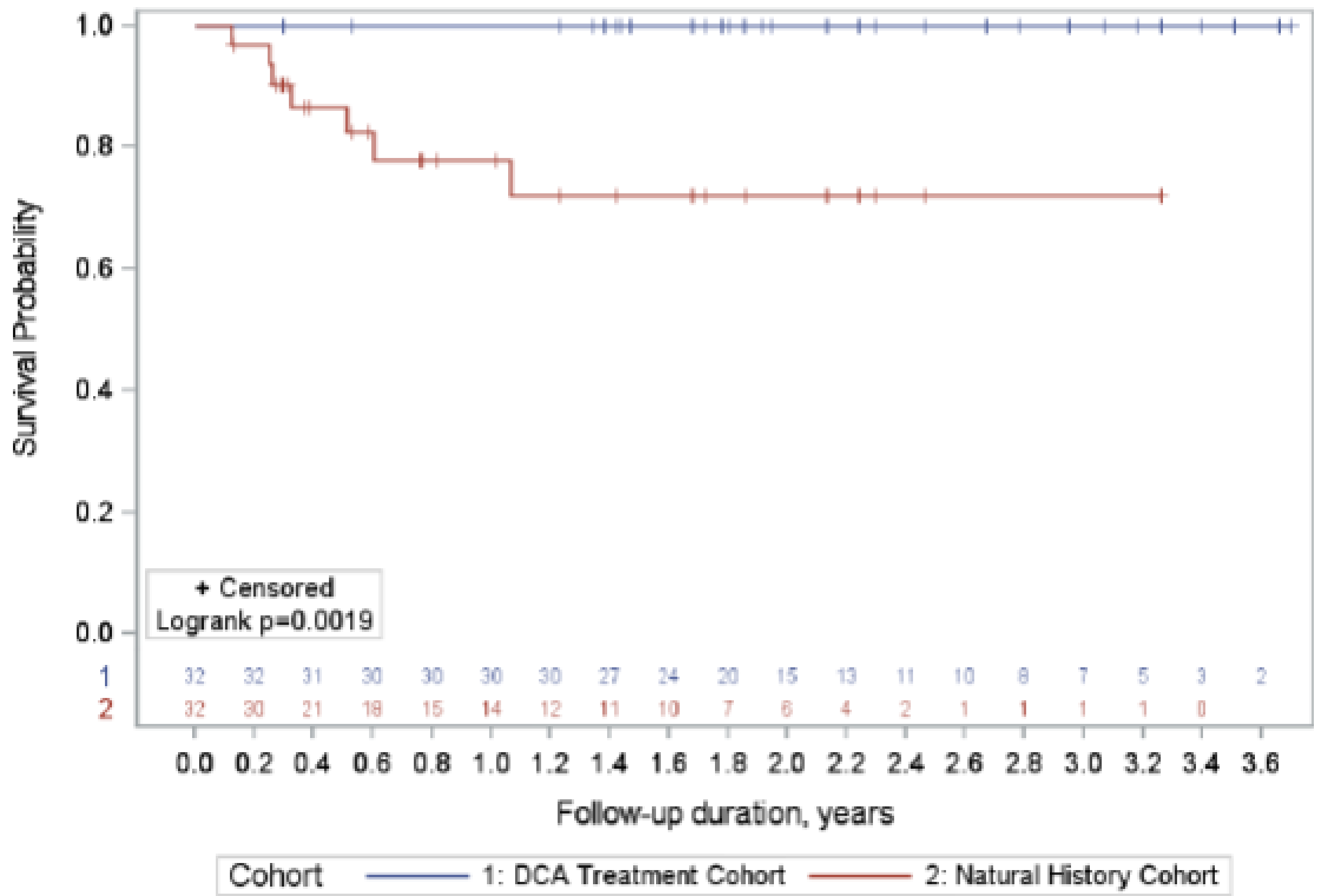

	DCA Treatment Cohort (N=32)	Natural History Cohort (N=32)
**Incidence Rate**		
Number of patients who died, n (%)	0 (0.0%)	7 (21.9%)
Total person-time in cohort, years	68.80	33.81
Events per 100 person-years (95% CI)	0.00 (0.00 - 4.35)	20.71 (8.32 - 42.66)

Figure S5. Overall Survival in Full Cohorts

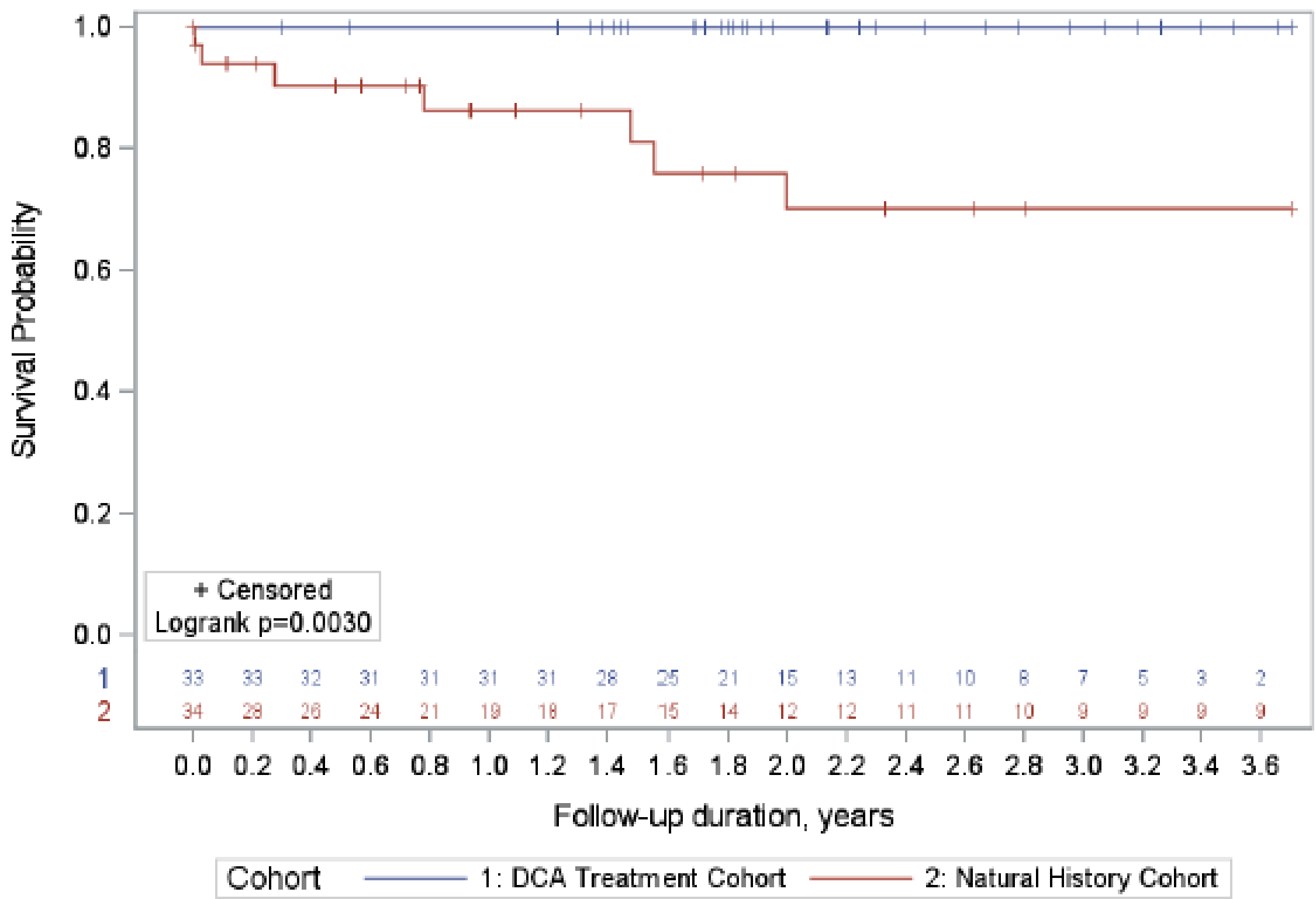

	DCA Treatment Cohort (N=33)	Natural History Cohort (N=34)
**Incidence Rate**		
Number of patients who died, n (%)	0 (0.0%)	7 (20.6%)
Total person-time in cohort, years	70.61	57.96
Events per 100 person-years (95% CI)	0.00 (0.00 - 4.24)	12.08 (4.86 - 24.88)

Figure S6. Overall Survival in the Sex- and Follow-up Duration-Matched Groups

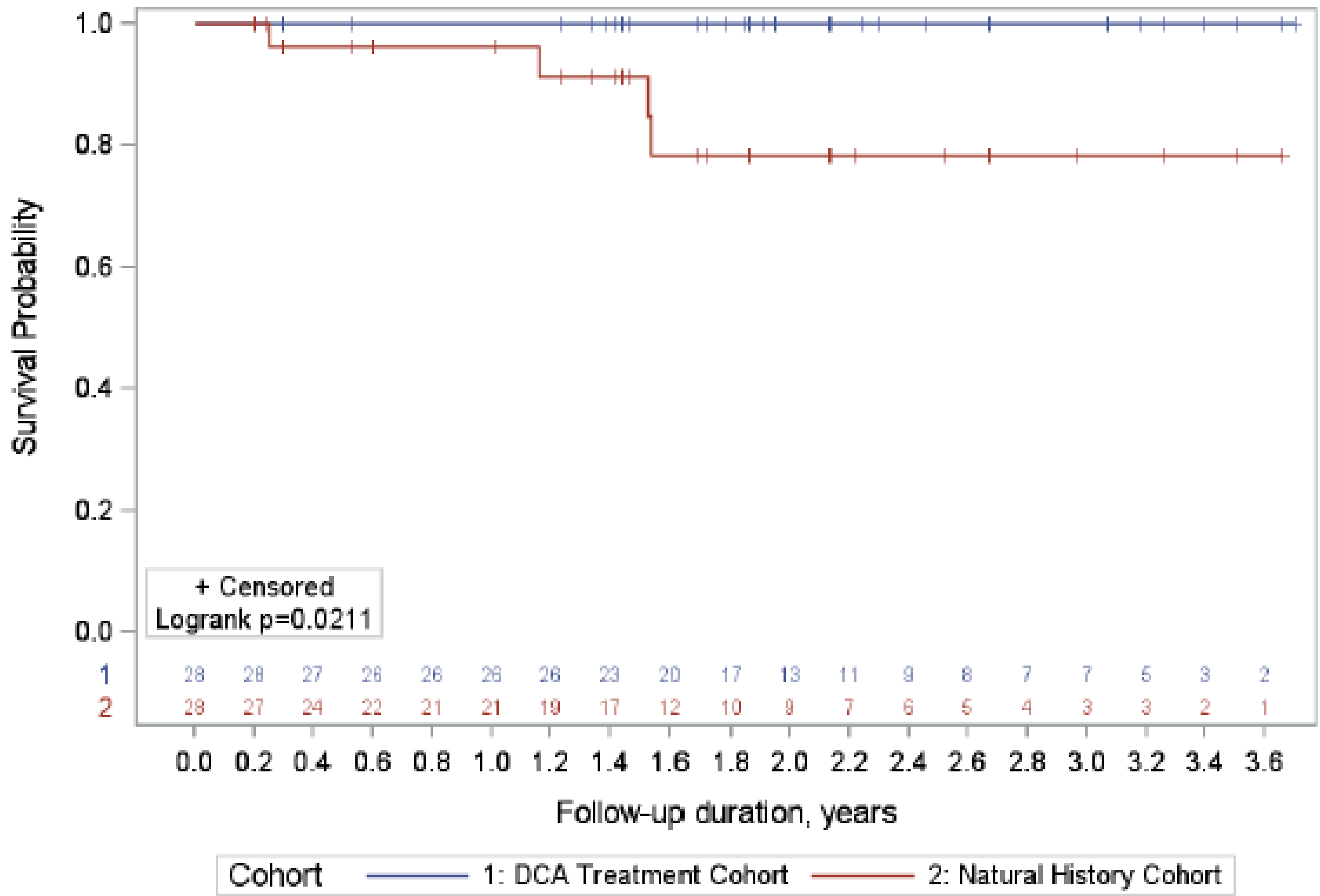

	DCA Treatment Cohort (N=28)	Natural History Cohort (N=28)
**Incidence Rate**		
Number of patients who died, n (%)	0 (0.0%)	4 (14.3%)
Total person-time in cohort, years	59.59	45.14
Events per 100 person-years (95% CI)	0.00 (0.00 - 5.03)	8.86 (2.41 - 22.69)

Figure S7. Overall Survival in the Sex- and Age-Matched Groups, Missing Age at Diagnosis Imputed to 0

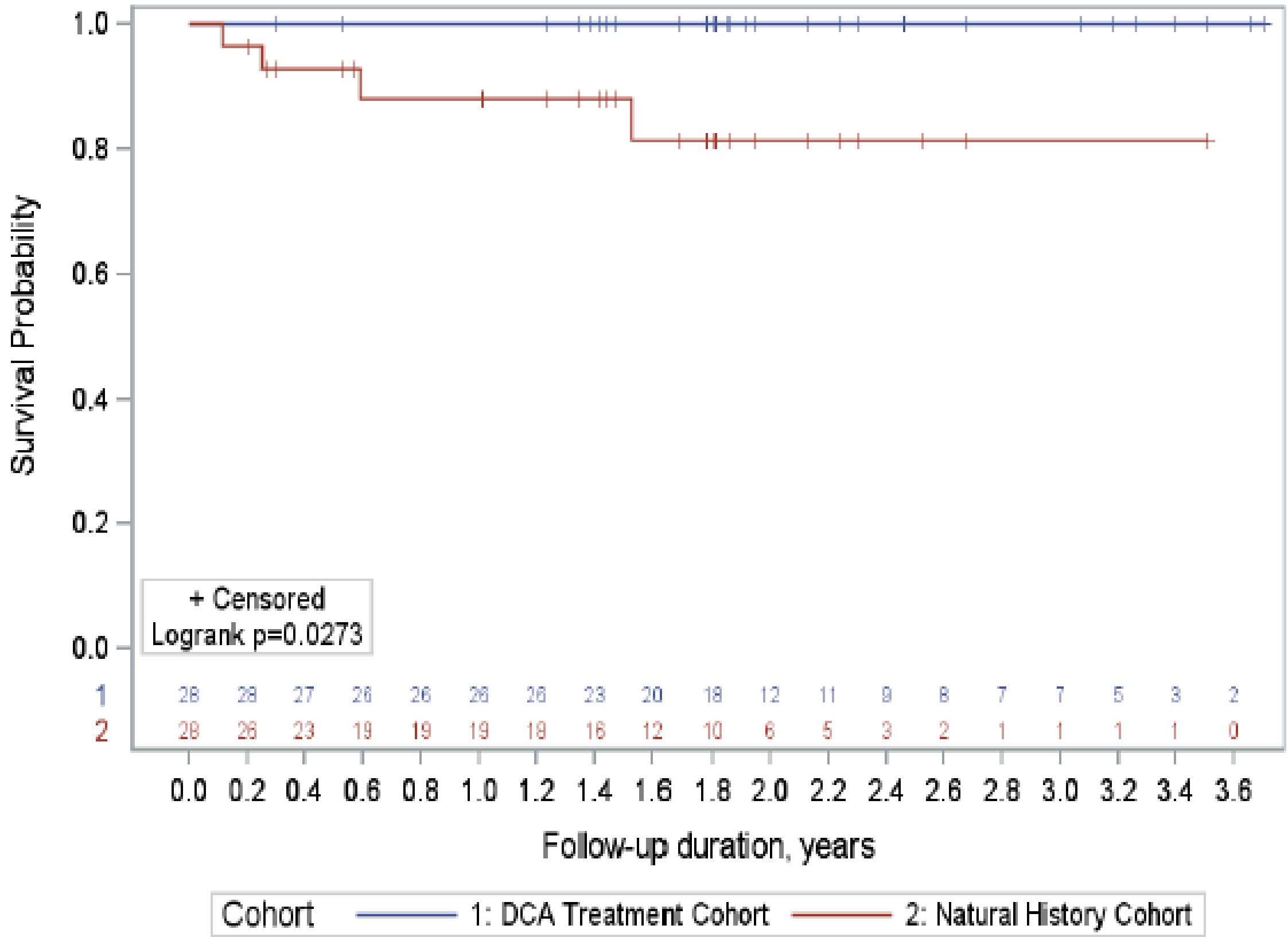

	DCA Treatment Cohort (N=28)	Natural History Cohort (N=28)
**Incidence Rate**		
Number of patients who died, n (%)	0 (0.0%)	4 (14.3%)
Total person-time in cohort, years	59.34	39.06
Events per 100 person-years (95% CI)	0.00 (0.00 - 5.05)	10.24 (2.79 - 26.22)

Figure S8. Overall Survival in the Sex- and Age-Matched Groups With Extended Follow-Up Time, Missing Age at Diagnosis Imputed to 0

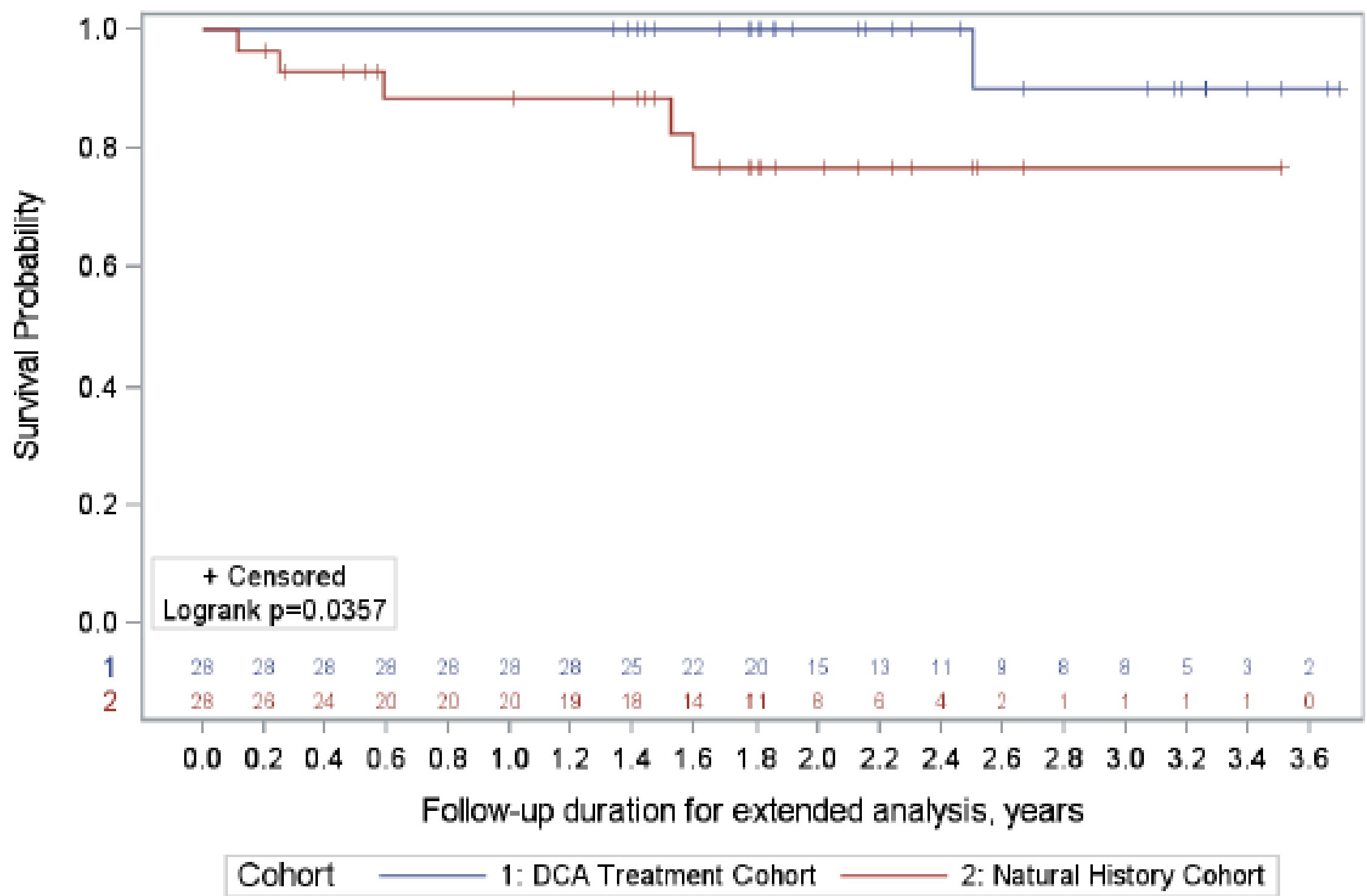

	DCA Treatment Cohort (N=28)	Natural History Cohort (N=28)
**Incidence Rate**		
Number of patients who died, n (%)	1 (3.6%)	5 (17.9%)
Total person-time in cohort, years	64.48	41.63
Events per 100 person-years (95% CI)	1.55 (0.04 - 8.64)	12.01 (3.90 - 28.03)

Figure S9. Overall Survival in the Sex- and Age-Matched Groups, Using Matching With Replacement

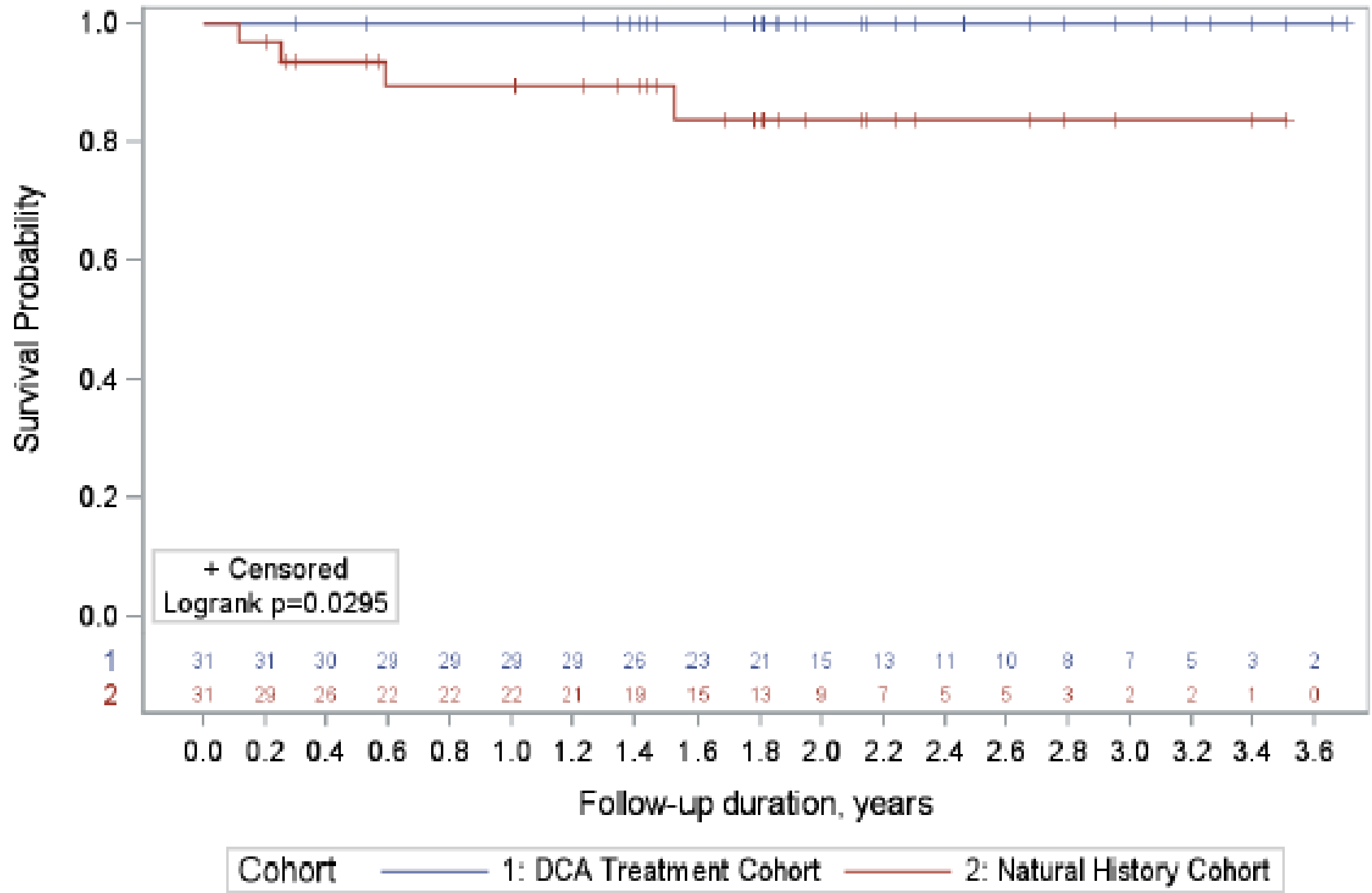

	DCA Treatment Cohort (N=31)	Natural History Cohort (N=31)
**Incidence Rate**		
Number of patients who died, n (%)	0 (0.0%)	4 (12.9%)
Total person-time in cohort, years	67.21	47.81
Events per 100 person-years (95% CI)	0.00 (0.00 - 4.46)	8.37 (2.28 - 21.42)

Figure S10. Overall Survival in the Sex- and Age-Matched Groups With Extended Follow-up Time, Using Matching With Replacement

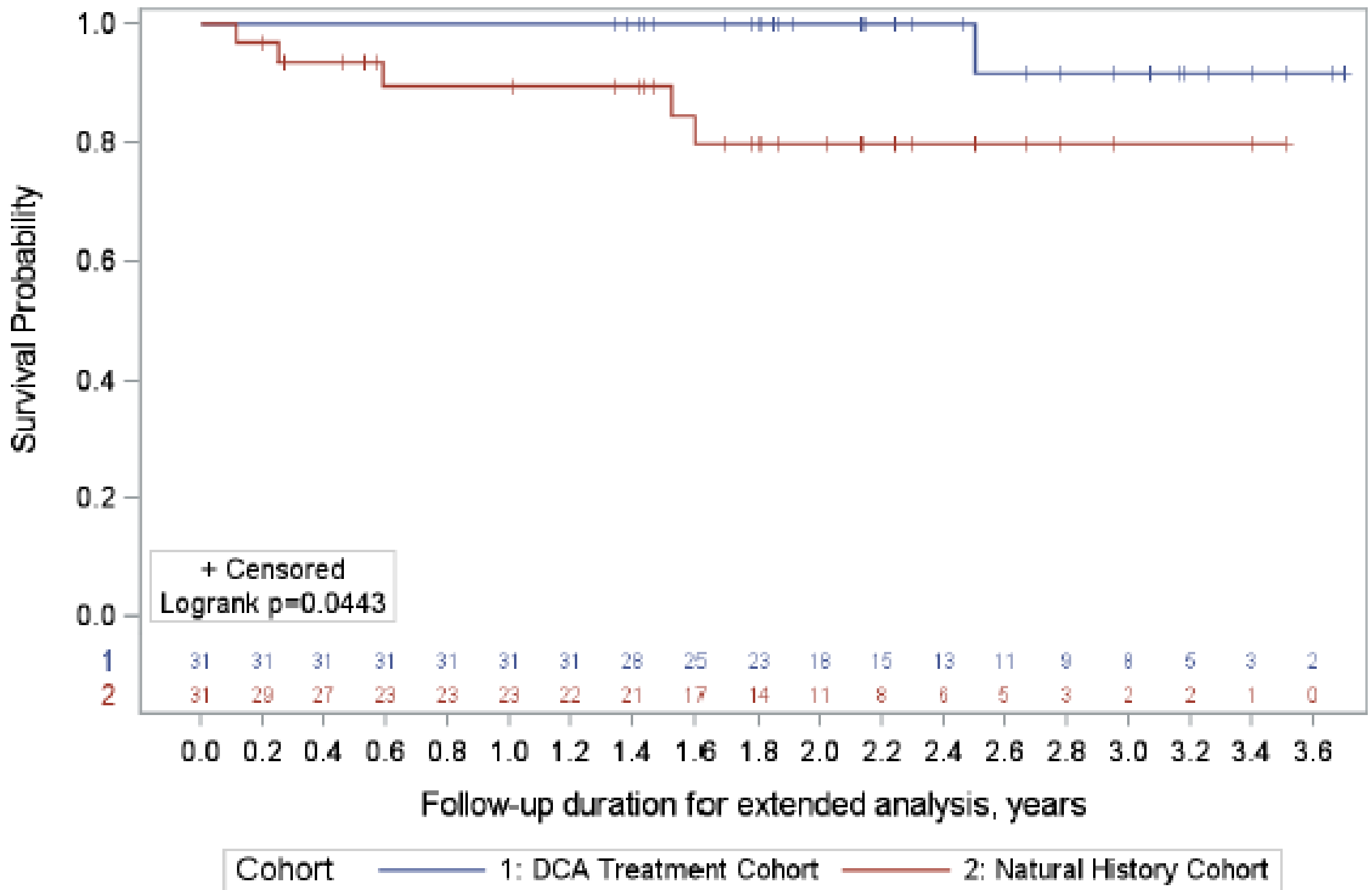

	DCA Treatment Cohort (N=31)	Natural History Cohort (N=31)
**Incidence Rate**		
Number of patients who died, n (%)	1 (3.2%)	5 (16.1%)
Total person-time in cohort, years	72.35	50.38
Events per 100 person-years (95% CI)	1.38 (0.03 - 7.70)	9.93 (3.22 - 23.16)

Extended follow-up time includes post-study discontinuation for the DCA treatment group
